# Public preferences for health data sharing governance: a discrete choice experiment in China

**DOI:** 10.3389/fpubh.2026.1883099

**Published:** 2026-10-20

**Authors:** Jie Li, Yulu Jin, Huaicheng Hui, Xisheng Huang

**Affiliations:** 1 School of Law, Chongqing University, Chongqing, China; 2 School of Law, Beijing Jiaotong University, Beijing, China; 3 School of Law, Tsinghua University, Beijing, China; 4 School of Law, Gansu University of Political Science and Law, Lanzhou, China

**Keywords:** China, data sensitivity, discrete choice experiment, health data governance, health data sharing, institutional trust, preference heterogeneity

## Abstract

**Background:**

Public acceptance is central to legitimate and effective health data sharing governance, yet evidence on how people evaluate multidimensional arrangements across data-sensitivity contexts remains limited. Existing studies often examine institutional attributes in isolation. We investigated public preferences for health data sharing governance in China and examined whether data sensitivity, trust in government organizations, and individual heterogeneity shaped these preferences.

**Methods:**

We conducted a discrete choice experiment with adult Chinese citizens using a D-efficient design. Fifteen choice sets were divided into three blocks, and each respondent was randomly assigned to one block. The experiment varied five attributes: data custodian, purpose of use, rights protection, incentive type, and data type. Using 7,405 valid choice observations from 1,481 respondents, we estimated average marginal component effects with ordinary least squares and respondent-clustered standard errors. Interaction models examined moderation by data type and by trust in government organizations, and a supplementary latent class analysis examined preference heterogeneity.

**Results:**

Within this design, data type had the largest average marginal component effect and moderated the effects of the other governance arrangements. For sensitive health data, respondents preferred strong rights protection and public hospital custodianship. For general health data, government-affiliated neutral third-party custodianship and non-cash incentives increased the probability of choosing a sharing profile, whereas the same arrangements had negative effects for sensitive data. The moderating role of trust in government organizations was conditional. After correction for multiple comparisons, higher trust strengthened the preference for government-affiliated custodians and weakened the effect of health points, and no other attribute effect varied with trust. A supplementary penalized latent class analysis suggested three exploratory preference profiles.

**Conclusion:**

Preferences in this online sample were context-dependent and are unlikely to be accommodated by uniform governance frameworks. Risk-stratified governance should combine stronger rights protection and accountable custodianship for sensitive data with streamlined procedures and calibrated incentives for general data. Trust in government organizations conditioned selected governance preferences, and the social usefulness of health information depends on the conditions under which it is collected, accessed, and shared.

## 1 Introduction

The rapid expansion of digital health systems has transformed personal medical data into a core resource for precision medicine, public health surveillance, and biomedical research (1–3). Alongside these opportunities, governance questions about how health data can be shared responsibly have become increasingly pressing for policymakers, health systems, and the public alike (4, 5). How to advance data utilization while protecting individual privacy has emerged as one of the defining challenges of contemporary health governance, made more acute by growing recognition that public legitimacy is a precondition for any sustainable data-sharing framework (6). Evidence consistently shows that the public does not broadly oppose secondary use of health data, but that support is conditional rather than unconditional (7, 8). Public attitudes vary with the sensitivity of the data concerned, the identity of the receiving organization, and the transparency of the governance arrangements in place (7). Privacy protection, informed consent, and clarity of purpose have been identified as critical requirements for acceptance across different national and institutional settings (8).

This issue is particularly significant in the Chinese context. In recent years, building on over a decade of ambitious health system reform that has substantially expanded coverage and reshaped the organization of care delivery (9), China has simultaneously accelerated its development of digital medicine and health data governance while progressively strengthening legal protections for sensitive personal information. Scholars have noted that China’s health data governance reflects a dual logic of “sharing” and “protection” operating in parallel. On the one hand, health data is assigned an important role in improving the efficiency of healthcare delivery, driving scientific innovation, and strengthening public health governance; on the other hand, concerns over privacy breaches, data misuse, and inadequate rights protection have been rising in tandem (10, 11). The challenge facing China, then, is not whether to advance health data sharing, but how to do so through institutional arrangements that the public finds acceptable. In particular, when confronted with concrete sharing schemes, what the public actually prioritizes in practice, including who holds custodianship, what purpose is served, whether sufficient control is retained, whether compensation is received, and how sensitive the data itself is, remains in need of further empirical investigation.

Research in this area has advanced along three broad lines. The first draws on privacy calculus theory, which holds that individuals weigh potential benefits against privacy risks when deciding whether to share data; support is higher when sharing is seen as generating research value, public benefit, or personal return (12–14). More recent work in this vein has moved toward context-sensitive analysis. Research on location-based services first demonstrated that privacy calculus operates through push-pull dynamics in which service benefits and perceived privacy risks interact in ways shaped by the specific context of disclosure (15), and subsequent empirical work has extended this framework to health data settings, attending to the moderating effects of perceived data control, incentive types, and data type variation on sharing willingness (16, 17). The second, drawing on institutional trust, social license, and governance legitimacy frameworks, argues that public support for data sharing depends substantially on trust in the data recipient, the governance process, and the underlying institutional arrangements (18–20). The third strand focuses on the governance architecture of specific sharing arrangements and the contextualized preferences these elicit from the public. Foundational work on the secondary use of health data identified the normative and operational parameters within which sharing preferences are formed (21), and subsequent experimental and survey research has confirmed that public support is neither abstract nor stable but varies systematically with data type, purpose of use, recipient entity, consent modality, and control rights design (22, 23). Complementing this, scholarship that reconceptualizes the public as active data producers has shown that patients develop nuanced understandings of how their records may be repurposed, and that these understandings shape acceptance in ways that aggregate survey measures may understate (24).

Taken together, these bodies of work provide important foundations, yet two gaps remain with direct implications for policy design. First, while existing studies have identified a range of relevant governance features, most treat these features as independent variables and assess their importance separately. This approach does not reflect how people actually evaluate data-sharing proposals, which present multiple institutional elements simultaneously. How the influence of any one feature shifts depending on the broader arrangement, and in particular on the sensitivity of the data at stake, remains underexplored (22, 23, 25). Second, in governance systems where government plays a central role, public trust in government organizations may condition how people respond to institutional arrangements, including choices about custodianship and incentive design. This interaction has received little direct empirical attention in the Chinese context, where government involvement in health data governance is both pervasive and consequential (10, 11, 26).

This paper examines how the public evaluates medical data-sharing arrangements when they are presented as complete governance proposals rather than isolated attributes. Using a discrete choice experiment with Chinese citizens, we estimate how four institutional features (custodial entity, purpose of use, rights protection, and incentive type) shape public acceptance of data-sharing schemes under two data-type contexts (general vs. sensitive health information). We also ask whether the data-type context modifies the effects of these institutional features, and whether trust in government organizations conditions how people respond to these arrangements. The aim is to generate evidence that can directly inform the design of more public-acceptable health data governance. The analytical approach draws on conjoint analysis and discrete choice experimentation, a methodological tradition with an established record in health economics for eliciting multidimensional preferences across competing attributes (27–29). Experimental designs are constructed in accordance with best-practice guidelines that balance statistical efficiency with respondent burden (30), and preference estimates are interpreted within the canonical framework for causal inference in conjoint analysis (31).

Within this design, data type carried the largest average marginal component effect, and general health information was markedly more acceptable than sensitive health information. On average across the two data types, government-affiliated neutral third parties, strong rights protection, health points and cash incentives increased the probability of choosing a sharing profile, whereas use for individual diagnosis and treatment reduced it. These effects varied with data type. For sensitive data, strong rights protection carried substantially greater weight, the government-affiliated third party became less acceptable than a public medical institution, and health points and honorary recognition reduced acceptance. Trust in government organizations played a narrower role. After correction for multiple comparisons, higher trust strengthened the preference for government-affiliated custodians and weakened the effect of health points, and the effects of the other governance attributes did not vary with trust.

The paper contributes to scholarship and to policy practice in three ways. First, it shows that data sensitivity is not simply one attribute among many: it serves as the risk context through which people evaluate all other governance features. The same institutional design may be acceptable for general health data but rejected when more sensitive data are involved. Second, it shows that trust in government organizations conditions a limited set of governance preferences, strengthening the preference for government-affiliated custodians and weakening the appeal of health points while leaving the other attribute effects unchanged. Third, it provides granular evidence to support more differentiated governance design. Rather than applying a single institutional template across data types, governance frameworks should be calibrated to the sensitivity of the data at stake: robust rights protections and clear accountability matter most for sensitive data, while incentives and neutral custodial arrangements carry more weight for general data. These findings are relevant to any health system working to build public-acceptable frameworks for health data governance. In doing so, this study also engages with the broader question of what makes health information useful for governance, a concern central to the Measurement for Change approach, by demonstrating that useful information in this domain must be conditional, contextually grounded, and attentive to the heterogeneity of public preferences rather than reducible to aggregate measures of acceptance.

## 2 Theoretical foundation and research hypotheses

### 2.1 Theoretical framework for analyzing public preferences on medical and health data sharing

Public judgments about health data sharing are formed under uncertainty. Once data enter the cycle of sharing and secondary use, the pathways they follow, the purposes they serve, the risk of re-identification and the availability of redress lie largely beyond what individuals can observe. Contextual integrity explains why the same information flow can appear legitimate in one governance setting and objectionable in another, since people judge flows against the norms of the context in which the data were first disclosed (32). Privacy calculus theory gives the complementary account of how the judgment is made. The theory originated in research on how individuals evaluate organizational practices for collecting and using personal information (13, 33) and holds that disclosure becomes more likely when perceived benefits offset perceived privacy risks (34). Later work showed that the nature of the data and the character of the recipient moderate this calculus (35, 36). In health data settings the weighing rarely proceeds through a full enumeration of consequences. People rely on visible signals about who holds the data, what the data will be used for, how much control they retain and whether participation brings any return (22). Cross-national discrete choice experiments show that these signals form a multidimensional preference structure in which recipient identity, purpose and oversight each have separate and interacting effects on acceptability (37).

The analytical framework links three elements, namely institutional attributes, data sensitivity and trust in government organizations. Institutional attributes are the features of a sharing arrangement that policy can set directly. The four attributes examined here are the data custodian, the purpose of use, rights protection and the form of incentive, which together determine how credible, legitimate and controllable an arrangement appears. Data sensitivity defines the risk context in which these attributes are evaluated. Data types differ in identifiability, stigmatization risk and the potential magnitude of harm, and highly sensitive data such as genetic information elicit more cautious attitudes together with stricter demands for withdrawal, de-identification and limits on commercial use (38). Trust in government organizations occupies a different position. Data type is a property of the arrangement, whereas trust is a disposition that respondents bring to the evaluation and that shapes how the provisions of an arrangement are read. In China the government is the principal rule-maker and also a central organizer and endorser of health data governance arrangements, so beliefs about the competence, benevolence and integrity of government organizations are likely to enter assessments of specific provisions (18). Trust in health information sharing systems is a recurrent determinant of public acceptance (19, 39). Research on social license adds that endorsement depends on confidence that governance authorities will honor public-value commitments and manage risks (40), and that endorsement can collapse quickly when governance is perceived as opaque or unaccountable (41).

The framework leads to three expectations about stated choices. The four institutional attributes shape the acceptability of a sharing proposal (H1–H4). Data sensitivity may change the weight and even the direction of these attribute effects (RQ1). Trust in government organizations may condition the same effects at the respondent level (RQ2). Data type was also varied experimentally as a fifth attribute, so its main effect is estimated alongside those of the four institutional attributes (Section 3.2).

### 2.2 Core institutional conditions and public sharing preferences

The four institutional attributes correspond to the questions people most often ask of a sharing proposal, namely who receives and holds the data, for what purpose, whether individuals keep meaningful control, and whether participation brings a return. Public acceptance of health data sharing varies systematically with each of these arrangements (42, 43), and acceptance of third-party use rests on an overall judgment of the recipient’s trustworthiness, public mission and capacity to bear responsibility (44).

#### 2.2.1 The data custodian and public sharing preferences

The identity of the custodian is among the most visible signals in a sharing arrangement, because the custodian determines who has access to the data, who bears custodial responsibility and who is accountable when harm occurs. Trust in the recipient and perceived accountability are among the strongest predictors of willingness to share across recipient types (45). The public is generally more comfortable with entities that have a strong public character, such as hospitals and research institutions, than with commercial entities or recipients whose boundaries of use are unclear (22). Lower comfort with commercial use also reflects uncertainty about liability, purpose creep and profit motives (46). The custodian contrast in the present design lies between public medical institutions and government-affiliated neutral third parties. Both have a public character. Public medical institutions have a direct clinical relationship with data subjects and relatively clear lines of responsibility, while neutral third parties are separated from clinical activity, which may ease concerns about functional overreach and conflicts of interest. The following hypothesis is therefore proposed.

*H1*: The data custodian will significantly influence public preferences regarding medical and health data sharing arrangements.

#### 2.2.2 The purpose of use and public sharing preferences

The purpose of use is a central criterion of legitimacy. Even when the recipient is the same, purposes differ in perceived necessity and in the clarity of their boundaries. Positive attitudes toward the reuse of health data are typically conditional on the expectation that the data will serve the common good, and commercialization is the main source of resistance (47). Medical research attracts greater acceptance than commercial uses such as marketing (22), and public appraisal depends on the public character, transparency and controllability of specific uses (42). Purposes that appear to benefit the individual need not be more acceptable. Where medical care, public administration, insurance and commercial services may intersect, use for individual diagnosis and treatment can raise concerns about the expansion of data use. Accordingly, the second hypothesis is stated as follows.

*H2*: The purpose of use will significantly influence public preferences regarding medical and health data sharing arrangements.

#### 2.2.3 Rights protection and public sharing preferences

Rights protection addresses the concern that data, once shared, pass beyond the individual’s control. What matters to the public is whether concrete arrangements preserve informed consent, withdrawal, correction and the ability to track how data are used. Dynamic consent interfaces that let participants manage their consent over time respond directly to this concern and can increase trust and participation (48). Meta-consent extends the same logic by allowing standing preferences for whole classes of research use (49). Patients attach importance to having a say in research use of their records and are uncomfortable when access is granted without notification (50), and some authors argue that patients should hold ownership-like entitlements over their medical data (51). Trust in health information sharing systems is tied to the belief that one’s interests will be respected (19), and arrangements that allow ongoing communication and adjustment are preferred to one-time, irrevocable authorization (20). Stronger and more operational protections should make an arrangement appear more controllable, which leads to the third hypothesis.

*H3*: Rights protection will significantly influence public preferences regarding medical and health data sharing arrangements; relative to weaker safeguards, stronger and more operationally robust rights protections will increase public acceptance.

#### 2.2.4 The form of incentive and public sharing preferences

Incentives affect both the perceived personal benefit of participation and the perceived fairness of the exchange. Privacy calculus theory predicts that acceptance increases when participation yields perceptible benefits (34). Discrete choice experiments on health data sharing confirm that return arrangements matter, with their direction and size depending on whether the incentive is concrete, valuable and credible (23, 25). Responses to returns are also conditioned by information sensitivity and by the perceived trustworthiness of the recipient (52). Evidence from China indicates that perceived data control and delay discounting shape willingness to share different types of health data, and that people who value immediate control are less easily persuaded by deferred or non-monetary returns (17). Weak or purely symbolic incentives are unlikely to change preferences, and an incentive read as payment for accepting risk may even dampen acceptance. The fourth hypothesis is stated as follows.

*H4*: The form of incentive will significantly influence public preferences regarding medical and health data sharing arrangements; relative to the absence of incentives or purely symbolic incentives, incentives with clear and tangible value are more likely to increase public acceptance.

### 2.3 Data sensitivity as risk context: the moderating role of data type

The effects of the four institutional attributes are unlikely to be the same across data types. Genetic, mental health and reproductive health data are more easily linked to identity, more open to stigmatization and more consequential if misused than routine clinical records. Relative to general electronic health records, such data elicit more cautious attitudes and stricter protection requirements (38). The caution has a scientific basis, because even nominally anonymized genomic data carry substantial re-identification risk (53). Willingness to share genomic data also varies across governance contexts, which indicates that risk appraisal depends on both the properties of the data and the perceived quality of safeguards (54). The same arrangement may therefore be judged by different standards when sensitive data are at stake.

Each institutional attribute may be affected, although the plausible direction differs across attributes. Demands for strict oversight of recipient identity and accountability intensify when highly sensitive data are shared (55). A government-affiliated neutral third party may therefore appear more protective because the custodian is separated from clinical and commercial interests, or less accountable because the custodian is more distant from the data subject. Sensitive data are more directly linked to identity exposure and discrimination (53), so any ambiguity about the purpose of use may provoke stronger resistance. Irreversible or untraceable uses of sensitive data carry greater potential for harm, so the weight attached to withdrawal rights and oversight of each use is likely to rise with sensitivity (48). Incentives may compensate for the higher perceived risk of sharing sensitive data, or they may be read as a signal that the risk is substantial, so their effect could strengthen or weaken as sensitivity increases (23, 25).

Existing evidence shows that the conditions attached to public support become more demanding for sensitive data (56), and data protection law codifies this heightened scrutiny by treating genetic data as a special category (57). The evidence does not establish the direction of the interaction for each attribute, and for the custodian and incentive attributes the arguments above point in opposite directions. Because the direction of these effects was not established *a priori* and the study was not preregistered, the moderating role of data type is stated as an exploratory research question rather than as a set of directional hypotheses.

*RQ1*: Does data type moderate the effects of the data custodian, purpose of use, rights protection and form of incentive on public preferences for health data sharing arrangements?

### 2.4 The moderating role of trust in government organizations

Trust in government organizations shapes how people interpret what a sharing arrangement offers. Health data sharing is embedded in governance systems that reflect broader settlements about data ownership, public value and the scope of institutional authority (6). In China the government makes the rules and also organizes, promotes and endorses many data governance arrangements, and willingness to share health data varies with perceptions of government trustworthiness, capacity and accountability (10, 26). Organizational trust comprises assessments of a trustee’s ability, benevolence and integrity (18), and initial trust is shaped by structural assurances and institutional cues even without prior direct experience (58, 59).

Trust may condition attribute effects in two ways. When people cannot verify whether governance commitments will be kept, existing trust serves as a prior for interpreting specific features of the system (60). Acceptance of data-intensive health research accordingly depends on confidence in the public-value orientation and risk-management capacity of the governance system (40), and cross-jurisdictional analyses identify transparency and public accountability as core requirements for such research (61). Trust may also change how much people rely on explicit safeguards and returns. Respondents with lower trust may lean more heavily on visible arrangements such as rights protection and incentives to reduce uncertainty, whereas respondents with higher trust may extend greater credit to government-affiliated custodians and government-endorsed purposes.

Existing research supports the general proposition that trust affects acceptance of health data governance, but evidence on how trust modifies the effects of specific institutional attributes is limited, particularly in the Chinese context. The moderating role of trust is therefore also stated as an exploratory research question.

*RQ2*: Does trust in government organizations moderate the effects of the data custodian, purpose of use, rights protection and form of incentive on public preferences for health data sharing arrangements?

## 3 Materials and methods

### 3.1 Study design

This study combines a discrete choice experiment (DCE) with a structured questionnaire survey to examine public preferences for alternative medical data-sharing arrangements and the mechanisms shaping those preferences. Compared with relying solely on attitudinal scales, a DCE presents respondents with choice tasks defined by multiple attributes and attribute levels, thereby identifying the relative influence of different design features on data-sharing decisions. Because medical data sharing simultaneously involves privacy risks, governance arrangements, and incentive structures, this approach is particularly well suited to analyzing how individuals make trade-offs in complex policy settings, and it has been widely used in health policy research to study preferences, including preferences regarding health data sharing (23, 25).

The study was conducted in two stages. The first stage consisted of a pilot study, which was used to assess the comprehensibility and feasibility of the questionnaire wording, scale items, and DCE tasks, and to determine whether the instrument required revision. The second stage consisted of the main survey. The integrated questionnaire included DCE choice tasks, a scale of trust in government organizations, a scale of willingness to share, and sociodemographic items. These data were used to estimate the average preference effects of key attributes of medical data sharing, to examine preference heterogeneity associated with data type, trust in government organizations, and respondent subgroups, and to assess the robustness of the main findings.

### 3.2 Attribute and experimental design

The selection of attributes and levels was grounded in the preceding theoretical analysis and the existing literature, and focused on the core considerations shaping public decision making on medical data sharing (22). These considerations are organized along two analytical layers: four institutional design dimensions that policy actors can directly manipulate—the data custodian, purpose of use, rights protection, and financial incentives—and one risk-context dimension, the type of data (general vs. sensitive health information), which is not itself a policy lever but constitutes the baseline signal of perceived risk against which institutional arrangements are evaluated. Specifically, the data custodian reflects public judgments about institutional trust and governance responsibility; the purpose of use corresponds to the perceived normative legitimacy of data sharing; rights protection captures individuals’ concerns about autonomy and control; and financial incentives reflect expectations of value compensation and fair exchange. Data type was experimentally varied as the fifth attribute in the D-efficient design, so its effect is identified within the prespecified choice model, conditional on the design and model assumptions, on the same basis as the four institutional attributes; conceptually, however, it is not a policy lever but the risk context against which those attributes are evaluated, and it is accordingly treated as a moderator in the analytical framework. Based on these considerations, a set of realistic and distinguishable levels was assigned to each attribute in order to approximate respondents’ decision making in real-world policy contexts. The attributes and corresponding levels are presented in Table 1.

**Table 1:** Attributes and levels included in the DCE.

Attribute	Levels
Data custodian	Public medical institutions; government-affiliated neutral third-party agency
Purpose of use	Medical research; infectious disease control; individual disease diagnosis and treatment
Rights protection	Basic protection; moderate protection; strong protection
Economic incentive arrangement	No incentive; honorary incentive; health points; cash incentive
Type of data	General health information; sensitive health information

Before the choice tasks, respondents read a description of each attribute and the following level definitions. Rights protection: basic protection, one-time notification as required by law, with authorization irrevocable once data are shared; moderate protection, authorization can be withdrawn at any time and an annual report on data use is provided; strong protection, in addition to moderate protection, the respondent’s confirmation is required before each use of the data and the data use is subject to regular third-party audit. Purpose of use: the overview table described the non-clinical public purpose as public health disease prevention, and the choice profiles labeled the same level infectious disease control (epidemic control in the tables and figures). Data type: general health information, routine medical information such as diagnostic records, test results and medication use; sensitive health information, highly private information such as genetic data, mental health records and history of infectious disease. Incentive: no incentive, purely voluntary contribution without any monetary or material return; health points, points redeemable for health services such as check-up packages or online consultation vouchers; cash payment, a fixed cash reward for the shared data (RMB 50 in the choice profiles), paid for example via WeChat or Alipay; honorary certificate, an electronic or paper certificate acknowledging the public-interest contribution.

A D-efficient design was used to generate the choice sets (62). The four institutional attributes together with the data-type context yielded 144 potential profiles in total. Presenting all possible combinations to respondents would have imposed substantial cognitive burden and undermined response quality. Therefore, under the constraint of questionnaire length, 15 choice sets were selected from the full set of possible combinations and divided into three blocks, with each block containing five choice tasks. Each task consisted of two alternatives, option A and option B, and respondents were asked to choose between them. No opt-out option was included, requiring respondents to make a forced choice between the two alternatives so that relative trade-offs across attribute levels could be identified more clearly. Existing studies have shown that the inclusion or exclusion of an opt-out option may affect respondents’ choice behavior as well as the estimated attribute effects and trade-offs (63, 64). Each respondent was randomly assigned to complete one block. This design reduced cognitive burden while preserving the statistical identification of attribute-level effects in the full sample. The design was generated in Python (NumPy) with a coordinate-exchange algorithm (3,000 iterations, fixed random seed) that minimized the D-error, det(X′X)^{−1/p}, evaluated under a null prior (*β* = 0); no commercial software or non-zero priors were used. The only constraint imposed was that the two alternatives within a set differ, and level balance, orthogonality, and the exclusion of dominated pairs were not imposed; the realized level balance across the design is reported in Supplementary Tables S1, S2. Within each profile the five attributes were displayed in a fixed order rather than randomized.

### 3.3 Questionnaire design and data collection

The questionnaire was administered in a fixed sequence. After providing informed consent, respondents first completed a scale measuring trust in government organizations, followed by an attention-check item. They then completed the discrete choice experiment, which comprised five binary choice tasks within a randomly assigned block; standardized explanations of the attributes and their meanings were presented before the choice tasks. Respondents subsequently completed a scale measuring willingness to share medical data, and finally provided sociodemographic information, including gender, age, educational attainment, income, place of residence, and recent medical service use. Because the trust scale was administered before the choice tasks, trust is treated as a pre-treatment respondent characteristic in the moderation analyses.

Data were collected through the Credamo platform. After completion of the pilot study (*N* = 151), the wording of the questionnaire and the formal survey instrument were reviewed against the pilot results, which indicated that no changes to the attributes, levels, or scale items were required. The main survey was conducted from March to April 2026 and targeted Chinese citizens aged 18 years and above. After reading the informed consent statement, respondents entered the questionnaire and were randomly assigned to one of the three blocks. Responses were screened sequentially. Questionnaires were excluded if the completion time was below 100 s, if the attention-check item was answered incorrectly, if responses were identical across all items of both attitudinal scales, or if any choice task was left unanswered. A total of 1,481 valid responses were retained for analysis.

### 3.4 Measures

The core dependent variable of this study was public preference for medical data-sharing arrangements. In the discrete choice experiment, this preference was measured by respondents’ actual choices between option A and option B in each choice task. Because each alternative consisted of different combinations of data custodian, purpose of use, rights protection, financial incentives, and type of data, respondents’ choices can be interpreted as expressions of their relative preferences for different institutional features and their combinations.

In addition to the contextualized choice tasks, the questionnaire included two attitudinal variables: trust in government organizations and general willingness to share. Trust in government organizations was measured primarily with reference to the classic dimensions of ability, benevolence, and integrity in the organizational trust literature, and was used to capture individuals’ overall trust in institutions involved in medical data governance (18). This scale consisted of 9 items. In the pilot study, the Cronbach’s α of this scale was 0.925, and in the main survey the Cronbach’s α of the overall scale was 0.872. Willingness to share medical data was used to measure respondents’ general attitudinal orientation toward medical data sharing, as distinct from their choices regarding specific hypothetical arrangements in the discrete choice experiment (26, 65). This scale consisted of 4 items. In the pilot study, its Cronbach’s α was 0.865, and in the main survey its Cronbach’s α was 0.822. In the formal analysis, trust in government organizations served as the respondent-level moderator.

In addition, the study collected sociodemographic variables, including gender, age, educational attainment, income, place of residence, and recent medical service use. These variables were used both to describe the sample characteristics and to support subsequent heterogeneity comparisons and robustness checks. By combining contextualized choice outcomes with attitudinal measures and sociodemographic information, the analysis is able to provide a more comprehensive account of the structure of public preferences for medical data sharing and the sources of variation in those preferences.

### 3.5 Statistical analysis

The statistical analysis comprised scale assessment, estimation of main effects, moderation analysis, and supplementary analyses of robustness and preference heterogeneity. First, the reliability and validity of the government organizational trust scale and the willingness to share medical data scale were assessed. Reliability was primarily evaluated using Cronbach’s alpha to examine internal consistency. Structural validity was tested through confirmatory factor analysis. Convergent validity and discriminant validity were assessed using the average variance extracted, composite reliability, and the heterotrait–monotrait ratio of correlations (HTMT), respectively.

Second, the main effects in the discrete choice experiment were estimated as average marginal component effects (AMCEs) using ordinary least squares regression. Because attribute levels were assigned by the experimental design independently of respondent characteristics, AMCEs can be estimated with a linear probability model (31); with a D-efficient rather than fully randomized design, this identification is conditional on the design and model assumptions. The dependent variable was respondents’ actual choice of alternatives, and the independent variables were the attribute levels, which were dummy coded against a reference level for each attribute. Standard errors were adjusted for clustering at the respondent level.

Based on the main effect estimation, moderation analyses were further conducted to examine whether attribute preferences varied by data type and government organizational trust. To assess the moderating role of data type, a fully interacted model including interactions between data type and all attribute levels was estimated, and the conditional average marginal component effects under different data-type conditions were reported. To assess the moderating role of government organizational trust, a continuous interaction model was estimated, supplemented by conditional average marginal component effects for higher- and lower-trust groups defined by a median split of the trust score (median = 5.889; higher-trust group *n* = 809, mean = 6.19; lower-trust group *n* = 672, mean = 5.23). The continuous interaction provides the primary inference, and the grouped estimates serve only for presentation. Because the lower-trust group also scored above the scale midpoint, the labels denote relative rather than absolute levels of trust. Because the interaction tests involved multiple comparisons, the Benjamini–Hochberg procedure was used to control the false discovery rate.

Supplementary analyses are reported in the Supplementary material. They comprised four robustness checks (a conditional logit specification, an alternative completion-time threshold, exclusion of the highly imbalanced choice set #13 as a leave-one-task-out sensitivity analysis, and adjustment for respondent covariates), a penalized latent class choice model (66), and subgroup comparisons by gender, age, education and trust level, with Benjamini–Hochberg correction applied to the subgroup tests.

## 4 Results

### 4.1 Sample characteristics and overview of measurement results

#### 4.1.1 Sample characteristics

A total of 1,481 valid questionnaires were obtained in the main survey. The basic characteristics of the sample are reported in Tables 2, 3. In terms of gender, 63.7% of respondents were female and 36.3% were male. The sample was concentrated in the 19–35 age range, with 32.5% aged 19–25 and 41.1% aged 26–35, together accounting for 73.6% of the sample. In terms of educational attainment, 91.0% of respondents held at least a bachelor’s degree, including 71.2% with a bachelor’s degree and 19.8% with a master’s degree or above. Urban residents accounted for 92.2% of the sample, whereas rural residents accounted for 7.8%. Monthly income was mainly concentrated in the RMB 3,001–10,000 range, with the largest group falling in the RMB 6,001–10,000 bracket (32.3%). In addition, 74.1% of respondents reported one to three healthcare visits in the previous 6 months.

**Table 2:** Sample characteristics: demographic profile (*N* = 1,481).

Variable	Category	*n* (%)
Gender	Female	943 (63.7)
	Male	538 (36.3)
Age	18 or below	4 (0.3)
	19–25	482 (32.5)
	26–35	608 (41.1)
	36–45	260 (17.6)
	46–55	76 (5.1)
	56 or above	51 (3.4)
Residence	Urban	1,366 (92.2)
	Rural	115 (7.8)
Education	Junior high or below	6 (0.4)
	Senior high / technical secondary	34 (2.3)
	Junior college	94 (6.3)
	Bachelor’s degree	1,054 (71.2)
	Master’s degree or above	293 (19.8)
Monthly income	RMB 3,000 or below	214 (14.4)
	RMB 3,001–6,000	316 (21.3)
	RMB 6,001–10,000	479 (32.3)
	RMB 10,001–15,000	276 (18.6)
	RMB 15,001 or above	196 (13.2)

**Table 3:** Sample characteristics: data relevance and healthcare utilization.

Variable	Category	*n* (%)
Occupational relevance to data	None	326 (22.0)
	Low	514 (34.7)
	Moderate	344 (23.2)
	High	268 (18.1)
	Very high	29 (2.0)
Healthcare visits in past 6 months	0	135 (9.1)
	1–3	1,097 (74.1)
	4–6	198 (13.4)
	7 or above	51 (3.4)

Overall, the sample was predominantly young, highly educated, and urban, with a relatively concentrated distribution in terms of age, education, and place of residence.

#### 4.1.2 Descriptive statistics of key variables

With regard to the main study variables, Table 4 summarizes the central tendencies of the trust and willingness-to-share scales and of the discrete choice experiment. The mean score for overall government organizational trust was 5.756 on a seven-point scale. The mean scores for the benevolence, competence, and integrity dimensions were 5.945, 5.625, and 5.699, respectively, indicating a moderately high level of trust in government organizations among respondents. The mean score for the overall willingness-to-share scale was 5.518, with item means ranging from 5.386 to 5.714, suggesting that respondents were generally supportive of medical and health data sharing, although such support was not unconditional.

**Table 4:** Descriptive statistics of key variables.

Variable	Indicator	Mean/percentage
Government organizational trust	Overall mean	5.756
	Benevolence mean	5.945
	Competence mean	5.625
	Integrity mean	5.699
Willingness to share	Overall mean	5.518
	Range of item means	5.386–5.714
Discrete choice experiment	Valid choice observations	7,405
	Range of option A choice shares	16.0–92.7%
Distribution of selected profile attributes	General health information	68.1%
	Government-affiliated neutral third party	51.7%
	Strong protection	39.3%
	RMB 50 cash reward	28.2%
	Health points	29.8%

The discrete choice experiment generated 7,405 valid choice observations (1,481 respondents × 5 tasks). Across choice sets, the share of respondents selecting option A ranged from 16.0% (set #7) to 92.7% (set #13). Among all selected profiles, general health information appeared in 68.1% of chosen options, government-affiliated neutral third-party custodians in 51.7%, strong protection in 39.3%, and RMB 50 cash rewards and health points in 28.2 and 29.8%, respectively (see Table 4).

#### 4.1.3 Assessment of scale quality and experimental design

In the full sample, Cronbach’s alpha was 0.872 for the overall government organizational trust scale and 0.822 for the willingness-to-share scale, indicating acceptable internal consistency. For the three trust subdimensions, alpha coefficients were 0.657 for benevolence, 0.694 for competence, and 0.781 for integrity. Although the first two values fell slightly below 0.70, the overall scale showed satisfactory reliability and was retained for subsequent analysis. Confirmatory factor analysis further supported the two-factor structure comprising government organizational trust and willingness to share, with acceptable model fit ( $\chi^{2}/\mathrm{df}=4.04$ , CFI = 0.974, TLI = 0.968, RMSEA = 0.045). Standardized factor loadings ranged from 0.55 to 0.81 and were all statistically significant.

In terms of convergent and discriminant validity, the willingness-to-share scale yielded an AVE of 0.543 and a CR of 0.826, while the government organizational trust scale yielded an AVE of 0.439 and a CR of 0.874 (Table 5). Although the AVE for government organizational trust was slightly below the conventional threshold of 0.50, convergent validity remained acceptable given the CR above 0.70 and the significance of the factor loadings, following established criteria (67, 68). The HTMT value between the two constructs was 0.629, below the stricter threshold of 0.85, indicating satisfactory discriminant validity (69). Taken together, these results suggest that the measurement quality of the scales was adequate for the formal analyses.

**Table 5:** Measurement quality assessment.

Construct	Cronbach’s *α*	AVE	CR
Government organizational trust	0.872	0.439	0.874
Willingness to share	0.822	0.543	0.826

The confirmatory factor analysis for the two-factor model of government organizational trust and willingness to share yielded $\chi^{2}/\mathrm{df}=4.04$ , CFI = 0.974, TLI = 0.968, and RMSEA = 0.045. The HTMT value between the two constructs was 0.629.

With regard to experimental design quality, the DCE tasks did not exhibit widespread dominance problems. Although a small number of choice sets produced relatively high selection rates for option A, the overall distribution remained within an acceptable range, indicating that the choice tasks were able to differentiate respondents’ preferences effectively. Combined with the pretest evidence and the observed distribution of choices in the main survey, the DCE design can be considered appropriate in terms of attribute specification and task construction.

### 4.2 Overall structure of preferences for medical and health data sharing

To examine overall public preferences for different medical and health data-sharing arrangements, this study estimated Average Marginal Component Effects (AMCEs) using ordinary least squares (OLS). The dependent variable was the respondent’s choice between profile A and profile B in each task (coded 1 if profile A was chosen and 0 otherwise). The model included the four institutional attributes—data custodian, intended use, rights protection, and incentive format—together with the data-type context, and used dummy coding with a designated reference category for each factor; data type is examined separately as a contextual moderator in Section 4.3. Standard errors were clustered at the respondent level to account for within-respondent dependence across repeated choices. The main-effects model contained 14,810 option-level observations, corresponding to 1,481 respondents who each completed five paired choice tasks, and the model explained a modest but non-trivial share of the variance ( $R^{2}=0.1322$ ).

Within this design, data type carried the largest AMCE among the five attributes, with general health information increasing the probability of profile selection by 0.3205 relative to sensitive health information (*p* < 0.001). Table 6 reports the AMCE estimates for the four institutional attributes and the data-type context, and Figure 1 presents the same estimates as a forest plot in which attribute levels are ranked by effect size and 95% confidence intervals are displayed alongside the point estimates. Among the institutional conditions, incentive format showed relatively large effects: both a cash payment of RMB 50 and health points significantly increased the probability that a sharing profile would be selected (*β* = 0.1447 and *β* = 0.1292, respectively; both p<0.001 ), whereas honorary recognition was not statistically significant ( β=−0.0155,p=0.208 ). With respect to rights protection, strong protection significantly increased public acceptance compared with basic protection ( β=0.0779,p<0.001 ), while moderate protection did not reach statistical significance ( β=0.0030,p=0.803 ). For data custodianship, a government-affiliated neutral third party significantly increased the probability of profile selection relative to a public hospital ( β=0.0594,p<0.001 ). Regarding intended use, personal diagnosis and treatment significantly reduced the probability of profile selection relative to medical research ( β=−0.1030,p<0.001 ), whereas epidemic control did not differ significantly from medical research ( β=0.0082,p=0.434 ).

**Table 6:** Main effects of DCE attributes: AMCE estimates for the four institutional features and the data-type context.

Attribute	Level	AMCE	SE	95% CI	p
Custodian	Govt. third party	0.059	0.012	[0.036, 0.083]	<0.001
Intended use	Epidemic control	0.008	0.011	[−0.012, 0.029]	0.434
Intended use	Personal diagnosis	-0.103	0.011	[−0.125, −0.082]	<0.001
Rights protection	Moderate protection	0.003	0.012	[−0.021, 0.027]	0.803
Rights protection	Strong protection	0.078	0.012	[0.054, 0.102]	<0.001
Incentive format	Health points	0.129	0.013	[0.104, 0.154]	<0.001
Incentive format	RMB 50 cash	0.145	0.012	[0.122, 0.168]	<0.001
Incentive format	Honorary recognition	−0.016	0.012	[−0.040, 0.009]	0.208

AMCE = average marginal component effect. OLS estimates with respondent-level clustered standard errors. Data type was included as a main term in the choice model, its direct effect is reported in the text, and its moderating role is examined in Section 4.3.

**Figure 1:**
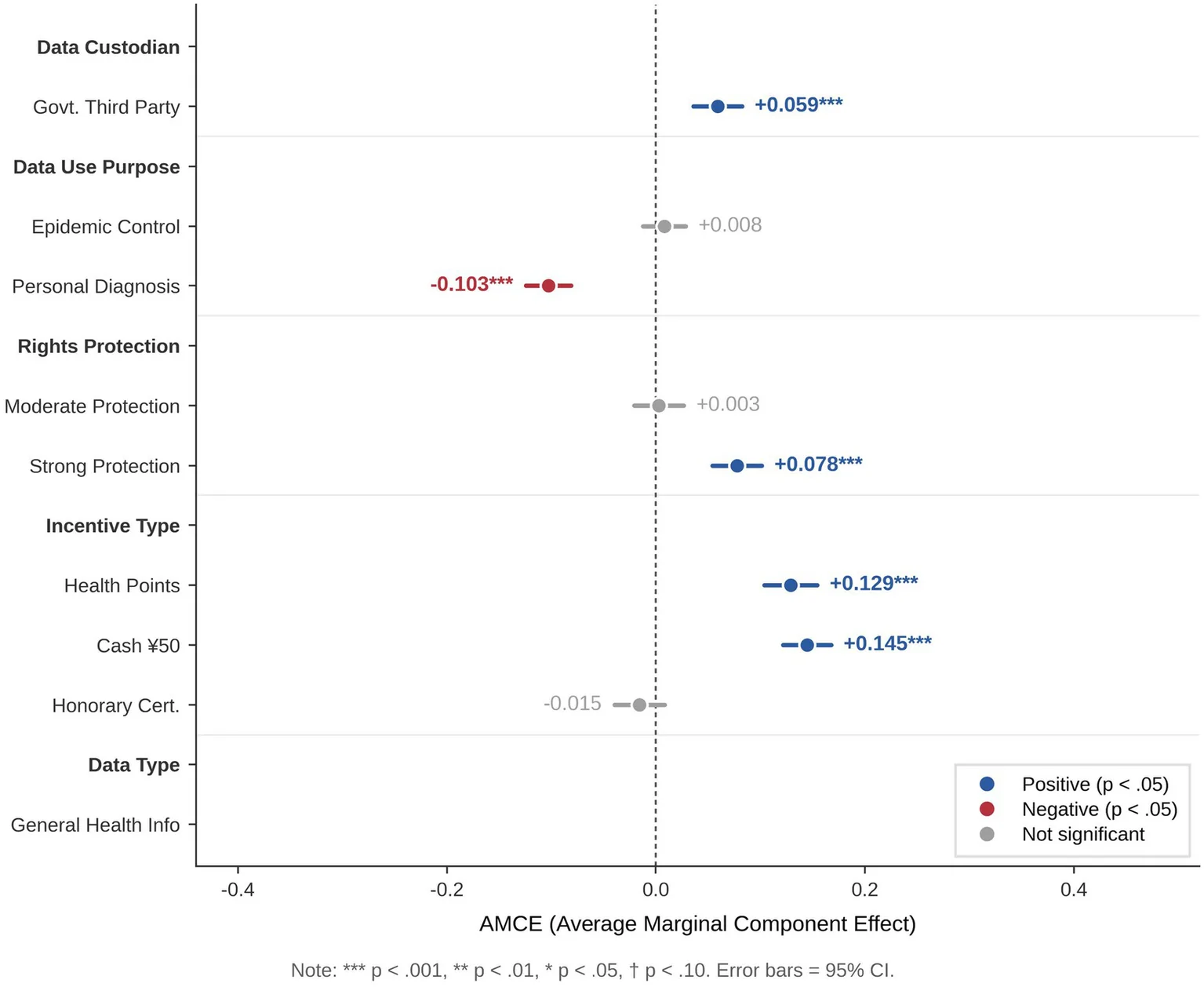
Average marginal component effects (AMCEs) of DCE attributes (four institutional features and the data-type context). Horizontal bars denote 95% confidence intervals based on respondent-clustered standard errors.

A visual inspection of Figure 1 reinforces this reading. The confidence intervals for RMB 50 cash, health points, and strong protection lie clearly to the right of zero and are visibly separated from the intervals of moderate protection and honorary recognition, which straddle the zero line. This visual separation, together with the ordering of point estimates, makes it easier to see that tangible incentives and strong rights protection dominate the institutional hierarchy, whereas symbolic incentives and moderate protection are statistically indistinguishable from their reference categories.

Taken together, these results suggest that public evaluations of medical and health data-sharing arrangements are shaped by multiple institutional features rather than by any single consideration. Within the set of institutional conditions examined here, incentive format and strong rights protection exhibited relatively pronounced positive associations with profile choice, while both data custodianship and intended use also significantly influenced public preference.

### 4.3 Moderating role of data type

Building on the main-effects model, a fully interacted specification was estimated to examine whether data type altered the effects of data custodian, intended use, rights protection, and incentive format on sharing preferences. Data type was coded as 0 for sensitive health information and 1 for general health information, and interaction terms were introduced between data type and each attribute level. The model was estimated using ordinary least squares (OLS) with respondent-level clustered standard errors. Because eight interaction terms were tested, the Benjamini–Hochberg procedure was applied to control the false discovery rate (FDR), and adjusted p values were used to assess interaction significance. To facilitate interpretation, conditional average marginal component effects (conditional AMCEs) were also reported separately for sensitive and general health information conditions.

The conditional AMCEs and interaction coefficients are reported in Table 7 and visualized in Figure 2, which places the sensitive and general health information conditions side by side so that sign changes and magnitude shifts can be read off directly from the panels.

**Table 7:** Moderating effects of data type on attribute preferences.

Attribute	Level	Sensitive	General	Interaction
Custodian	Govt. third party	−0.0478***	0.0803***	0.1281***
		(0.0135)	(0.0130)	(0.0103)
Use	Epidemic control	−0.0422**	−0.0027	0.0395**
		(0.0134)	(0.0144)	(0.0139)
Use	Personal diagnosis	−0.3333***	−0.1052***	0.2281***
		(0.0169)	(0.0141)	(0.0149)
Protection	Moderate	0.0370*	−0.0009	−0.0379**
		(0.0161)	(0.0166)	(0.0131)
Protection	Strong	0.2208***	−0.0212	−0.2420***
		(0.0139)	(0.0140)	(0.0148)
Incentive	Health points	−0.0296*	0.2436***	0.2732***
		(0.0141)	(0.0160)	(0.0156)
Incentive	RMB 50 cash	0.0756***	0.1794***	0.1038***
		(0.0165)	(0.0140)	(0.0189)
Incentive	Honorary recognition	−0.1096***	0.0578***	0.1673***
		(0.0161)	(0.0172)	(0.0187)

Conditional AMCEs were estimated using OLS with respondent-level clustered standard errors. Interaction p -values were adjusted using the Benjamini–Hochberg procedure. Sensitive = sensitive health information; General = general health information. Significance markers are based on raw p -values: *** p<0.001 , ** p<0.01 , * p<0.05 .

**Figure 2:**
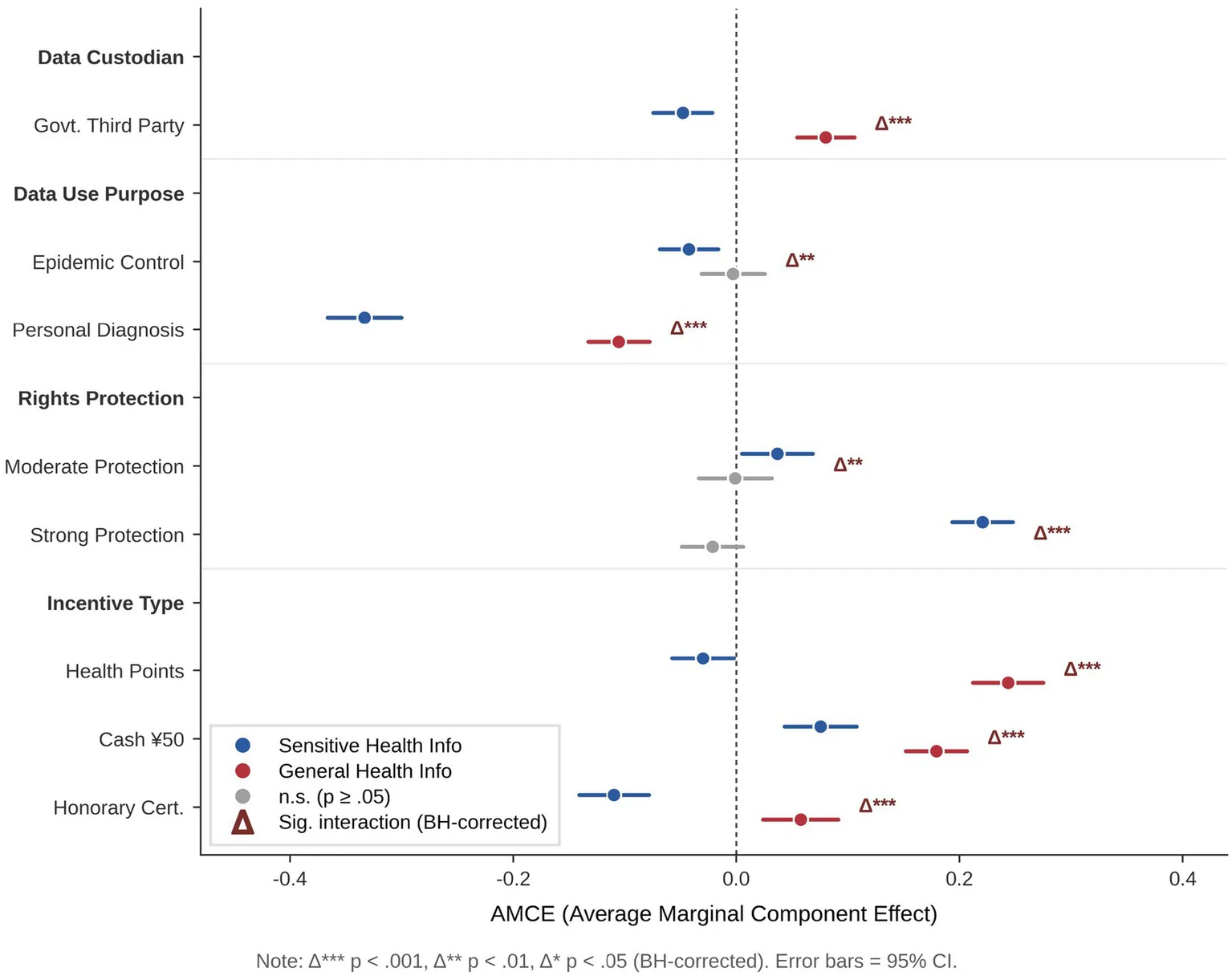
Conditional AMCEs under sensitive and general health information conditions. Each panel reports the effect of an attribute level, conditional on data type.

The results show that data type significantly moderated the effects of multiple institutional attributes. For data custodian, the effect of a government-affiliated neutral third party relative to a public hospital differed sharply across the two data conditions. Under sensitive health information, the effect was negative ( β=−0.0478 , p<0.001 ), whereas under general health information it became positive ( β= 00803, p<0.001 ). The interaction coefficient was 0.1281 and remained significant after BH correction ( p<0.001 ), indicating that data type altered both the direction and magnitude of the custodianship effect.

Data type also moderated the effects of intended use. For epidemic control relative to medical research, the estimated effect was negative under sensitive health information ( β=−0.0422 , p<0.001 ) but close to zero under general health information ( β=−0.0 027, n.s.), with an interaction coefficient of 0.0395 (BH-adjusted p= 0.0045). For personal diagnosis and treatment relative to medical research, the negative effect was substantially larger under sensitive health information ( β=−0.333 , p<0.001 ,) than under general health information ( β=−0.1052 , p<0.001 ), and the corresponding interaction coefficient was 0.2281 (BH-adjusted p<0.001 ).

The moderating role of data type was equally pronounced for rights protection. Strong protection showed a substantial positive effect under sensitive health information ( β=0.2208 , p<0.001 ), whereas the same level of protection no longer reached statistical significance under general health information ( β=−0.0212 , n.s.). The interaction term was negative and significant ( β=−0.2420 , BH-adjusted p<0.001 ). A similar pattern was observed for moderate protection, which showed a weak positive effect under sensitive health information ( β=0.0370 , p<0.05 ) but was close to zero under general health information ( β=−0.0009 , n.s.), with a significant interaction coefficient of −0.0379 (BH-adjusted p=0.0045 ).

The effects of incentive format also varied across data conditions. Health points had a negative effect under sensitive health information ( β=−0.0296 , p<0.05 ) but a strong positive effect under general health information ( β=0.2436,p<0.001 ), yielding an interaction coefficient of 0.2732 (BH-adjusted p<0.001 ). A cash payment of RMB 50 was positive in both conditions, although the magnitude was larger under general health information (sensitive: β=0.0756 , p<0.001 ; general: β=0.1794 , p<0.001 ; interaction: β=0. 1,038, BH-adjusted p<0.001 ). Honorary recognition was negative under sensitive health information ( β=−0.1096 , p<0.001 ) but positive under general health information ( β=0.0578 , p<0.001 ), with an interaction coefficient of 0.1673 (BH-adjusted p<0.001 ).

Taken together, these findings indicate that the data-type context was associated not only with direct differences in profile acceptance but also with significant changes in how the four institutional attributes operated. Across the two data contexts, the direction and magnitude of the effects of data custodian, intended use, rights protection, and incentive format all varied. Figure 2 makes this pattern particularly visible for custodianship, health points, and honorary recognition, where the conditional intervals lie on opposite sides of zero across the two data conditions, whereas cash incentives remain on the positive side in both panels but with clearly different magnitudes.

### 4.4 Moderating role of government organizational trust

To assess whether government organizational trust altered the effects of institutional attributes on sharing preferences, a continuous moderation model was estimated by interacting mean-centered trust with each attribute level. The model included the full set of DCE attributes, respondent-level government organizational trust, and all trust-by-attribute interaction terms. OLS was used for estimation, with standard errors clustered at the respondent level. Because nine interaction terms were tested, the Benjamini–Hochberg procedure was applied to control the false discovery rate (FDR). In addition, conditional AMCEs were reported for the higher- and lower-trust groups defined in Section 3.5 in order to facilitate interpretation of the interaction patterns.

Table 8 reports the interaction coefficients and BH-adjusted *p* values, and Figure 3 plots the marginal effect of selected attributes as a continuous function of trust, so that the direction of moderation can be read from the slopes. Grouped conditional AMCEs for the higher- and lower-trust groups are reported in Supplementary Table S13 and Supplementary Figure S1.

**Table 8:** Moderating effects of government organizational trust on attribute preferences.

Attribute	Level	Coefficient	SE	BH p
Custodian	Govt. third party	0.0511	0.0175	0.0302
Use	Epidemic control	−0.0049	0.0155	0.7520
Use	Personal diagnosis	−0.0132	0.0156	0.5984
Protection	Moderate	0.0100	0.0164	0.6980
Protection	Strong	−0.0165	0.0174	0.5984
Incentive	Health points	−0.0520	0.0192	0.0302
Incentive	RMB 50 cash	−0.0232	0.0186	0.4826
Incentive	Honorary recognition	−0.0062	0.0184	0.7520
Data type	General health information	−0.0351	0.0147	0.0519

OLS estimates with respondent-level clustered standard errors. BH-adjusted p -values are reported for the interaction terms.

**Figure 3:**
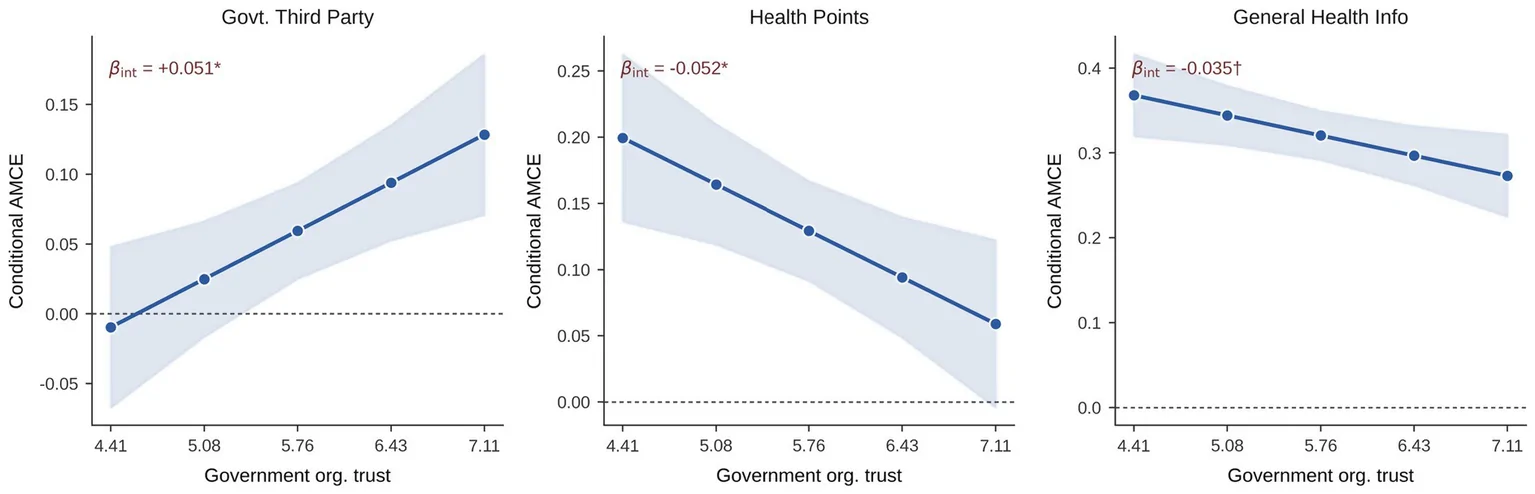
Marginal effects of selected attributes across levels of government organizational trust. Shaded bands represent 95% confidence intervals.

The results show that, after BH correction, significant moderation was observed for two attribute levels. First, government organizational trust positively moderated the effect of a government-affiliated neutral third party relative to a public hospital ( β=0.0511 , BH-adjusted p=0.0302 ). This indicates that the positive effect of a government-affiliated neutral third party became stronger as trust in government organizations increased. The grouped conditional AMCEs are consistent with this pattern: among higher-trust respondents, the effect of a government-affiliated neutral third party was 0.0691, compared with 0.0473 among lower-trust respondents. The custodian effect was positive in both groups, so trust changed its magnitude without reversing its direction.

Second, government organizational trust negatively moderated the effect of health points relative to no incentive ( β=−0.0520 , BH-adjusted p=0.0302 ). The grouped conditional AMCEs point in the same direction. The positive effect of health points was 0.1526 in the lower-trust group and 0.1101 in the higher-trust group, suggesting that the appeal of this incentive format weakened as trust increased.

These two patterns are also visible in Figure 3, where the slope for government-affiliated neutral third-party custody is upward and the slope for health points is downward as trust increases, whereas the slopes for most other attributes are approximately flat. The remaining interaction terms did not remain statistically significant after BH correction. No significant moderation was observed for epidemic control or personal diagnosis and treatment, for either moderate or strong rights protection, or for cash and honorary incentives. The interaction between government organizational trust and general health information was marginal in the unadjusted model but did not survive BH correction ( β=−0.0351 , BH-adjusted p=0.0519 ). Government organizational trust therefore had a conditional influence confined to two of the nine tested terms, data custodianship and health-point incentives, and most attribute effects were stable across the trust distribution (Figure 3).

### 4.5 Supplementary analyses

Four sensitivity analyses assessed the stability of the main effects (Supplementary Tables S21–S24). A conditional logit model estimated on the differenced attributes reproduced the sign of all nine attribute effects. Significance agreed for seven of the nine; epidemic control (*p* < 0.01) and moderate protection (*p* < 0.10) reached significance only in the conditional logit, and the approximate average marginal effects from the conditional logit were of the same order as, though generally smaller than, the OLS estimates. Raising the completion-time threshold from 100 to 160 s (*n* = 1,448) changed no coefficient by more than 0.0013. Excluding the highly imbalanced choice set #13 left eight of nine coefficients unchanged in sign, and the one sign change concerned epidemic control, which was non-significant in the baseline model. Adding respondent covariates (gender, age, education and government organizational trust) left the attribute effects virtually unchanged.

A penalized latent class logit model examined unobserved preference heterogeneity (Supplementary Tables S14, S15 and Supplementary Figure S2). Penalized (L2) estimation was required because the unpenalized likelihood does not attain a finite maximum with five choice tasks per respondent and nine class-specific parameters. Model selection was not decisive. Both BIC conventions marginally favored a four-class model, but that solution was recovered in only 2 of 50 random starts, against 9 of 50 for the three-class model, which is reported as the most stable interpretable summary. Average posterior assignment probabilities were 0.89, 0.86 and 0.85. The largest class (51.3%) responded positively to strong rights protection, health points and cash incentives (safeguard-and-incentive responsive). A second class (29.0%) responded negatively to most arrangements, most strongly to the individual diagnosis purpose (sensitivity-averse), and a third class (19.7%) had coefficients near zero on most attributes (low-engagement). These labels describe coefficient patterns and are not validated psychological constructs or fixed population segments. The class structure nonetheless shows that the aggregate AMCE averages over opposing preferences, as the sample-level effect of strong protection (0.078) lies between a strongly positive coefficient in the largest class (1.33) and a negative coefficient in the sensitivity-averse class (−1.49).

Subgroup analyses by gender, age, education and trust group showed limited observable heterogeneity (Supplementary Tables S16, S17 and Supplementary Figure S3). After BH correction, the only significant subgroup difference concerned data type, with a larger preference for general over sensitive health information among lower-trust than among higher-trust respondents (difference = −0.0668, BH-adjusted *p* = 0.022). The continuous trust × data type interaction in Table 8 pointed in the same direction but did not survive BH correction (BH-adjusted *p* = 0.052), so this difference is treated as suggestive. Gender differences for cash incentives and data type were marginal (BH-adjusted *p* = 0.054), and no difference by age or education survived correction.

## 5 Discussion

Data sensitivity substantially changed how respondents weighed governance arrangements, while trust in government organizations conditioned only a small subset of those trade-offs. The observed patterns are reported here separately from their possible explanations, because the design recorded stated choices among hypothetical profiles and did not measure perceived risk, accountability judgments or motivation. The mechanisms considered below are candidate explanations consistent with the results, and none of them was empirically verified. Privacy calculus has often been modeled as a stable trade-off between perceived risks and benefits (34, 70), whereas a growing body of evidence suggests that the trade-off is neither linear nor invariant across contexts (14).

### 5.1 Data sensitivity and the weighting of governance attributes

The clearest empirical result is the dependence of attribute effects on data type. For sensitive health data, a government-affiliated neutral third party reduced the probability of choosing a profile relative to a public hospital (*β* = −0.048), strong rights protection increased it (*β* = 0.221), and health points and honorary recognition reduced it (*β* = −0.030 and *β* = −0.110). For general health data, the third-party custodian (*β* = 0.080), health points (*β* = 0.244) and honorary recognition (*β* = 0.058) increased the probability of choice, while strong protection had no detectable effect (*β* = −0.021). Cash payment increased choice probability in both contexts, with a larger effect for general data, and the negative effect of the individual diagnosis purpose was about three times larger for sensitive data. These sign reversals are consistent with evidence that privacy decisions depend on data type and perceived harm (52, 71) and with recent findings that health data sharing decisions vary across digital health contexts (16).

Several explanations are compatible with the custodian reversal. Public hospitals have a direct clinical relationship with patients and more visible lines of accountability, and respondents may have preferred that proximity when the stakes were higher. A third party may also have added uncertainty about control and the attribution of responsibility for sensitive data. The reversal does not fit the assumption that intermediaries uniformly enhance trust (72, 73). Perceived accountability and institutional proximity were not measured, so these readings remain hypotheses for future research.

Strong protection mattered for sensitive data and had no detectable effect for general data. One possible explanation is that safeguards become salient mainly when perceived threat is high, as dual-calculus perspectives suggest (74). Respondents may also have regarded additional protections for routine data as unnecessary or procedurally burdensome, in line with evidence of diminishing returns to privacy safeguards (14). Neither perceived threat nor perceived burden was measured.

Health points and honorary recognition reduced the probability of choice for sensitive data and increased it for general data, and the difference for health points was the largest across the two conditions (Δβ = 0.273). Incentives offered for sensitive data may have been read as a signal of hidden risk or as compensation for potential harm, which could prompt distrust (14, 75). Incentives may also crowd out intrinsic motives such as altruism when privacy concerns are salient (76). Differential attention to attributes across risk conditions and social desirability pressures could produce the same pattern. The design cannot adjudicate among these explanations, and the result is best stated as a boundary condition, namely that the effect of incentives depended on data sensitivity.

### 5.2 The conditional role of trust in government organizations

Trust in government organizations moderated two of the nine tested terms after BH correction. Higher trust strengthened the preference for a government-affiliated neutral third party over a public hospital. The custodian effect was positive in both the higher- and lower-trust groups (0.069 and 0.047), so trust changed the magnitude of the effect without reversing its direction. Higher trust also weakened the effect of health points (0.110 and 0.153), while the effects of cash and honorary incentives, purposes of use and rights protection showed no significant trust moderation. Trust is therefore better described as conditioning selected governance preferences than as a general moderator of institutional attributes. A plausible reading is that respondents with greater confidence in government organizations extend more credit to government-affiliated custodians and rely less on returns to offset uncertainty, but this account was not tested. The trust distribution was concentrated toward the upper end of the scale, and the lower-trust group still scored above the midpoint, which limits inference about respondents with low trust.

### 5.3 Policy implications

The findings carry several implications for health data governance, with the caveat that they derive from stated choices in an online sample. First, governance arrangements could be differentiated by data sensitivity, since the same arrangement attracted opposite responses for sensitive and general data. Second, for sensitive data, strong rights protection, which added per-use confirmation and third-party audit to the right of withdrawal, together with custodianship by public healthcare providers, was most strongly associated with acceptance. Third, incentives such as health points were associated with higher acceptance only for general data and may be counterproductive where privacy concerns are salient. Fourth, because trust conditioned only the custodian and health-point effects, trust-building is unlikely to substitute for safeguards and matters chiefly where government-affiliated custodians are involved. As health data systems increasingly incorporate artificial intelligence and automated decision-making, effective governance must address not only privacy and data security but also accountability for system error, operational failure, and potential harm; these questions are especially acute in clinical and diagnostic settings, where failures involving such systems raise complex issues of professional liability, institutional responsibility, and patient safety (77).

### 5.4 What makes health information socially useful? Reflections through a measurement for change lens

What makes health information socially useful within data-sharing systems is a central question of the Measurement for Change (M4C) approach. The present findings suggest that the usefulness of health data depends on the governance context in which sharing occurs as well as on its scientific or public health potential. The same custodial and incentive arrangements raised acceptance for general health data and lowered it for sensitive data, so aggregate measures of acceptance would have concealed the conditions under which sharing is supported. Preference information that is conditional and heterogeneous corresponds to what M4C describes as the expansion of understanding and meaning, as distinct from the accumulation of data. The institutional features of health data governance are accordingly better assessed as components of a system that the public evaluates as a whole, in line with the Research Topic’s framing from silos to systems, and information generated from that perspective is more likely to serve as actionable knowledge for governance.

### 5.5 Limitations

Several limitations qualify the findings. First, the sample was drawn from the Credamo online panel without probability sampling and was predominantly young, urban and highly educated, and no population weights were applied because selection probabilities were unavailable. Recruitment through an online panel also introduces self-selection and possible professional-respondent and digital-literacy biases, which compound the demographic skew. The estimates therefore characterize the observed online sample and may not generalize to older, rural or less-educated adults in China. Second, forced binary choices identify relative preferences between displayed profiles and do not estimate opt-out behavior, absolute participation or real-world sharing decisions. Third, the level ‘individual disease diagnosis and treatment’ may have been interpreted as primary care rather than secondary use, so that coefficient is instrument-specific. Fourth, trust was self-reported and measured once, its distribution was negatively skewed and range-restricted, the median-split groups simplify a continuous construct, and the trust interactions do not establish trust as a causal driver of preferences. Fifth, the latent class solution required penalized estimation, the number of classes was not decisively determined, and the class labels are exploratory. Finally, the explanations offered for the coefficient reversals, including accountability concerns, risk signaling and motivational crowding, were not directly measured and should be tested in studies that measure perceived risk, accountability and trust in the specific custodian.

## Data Availability

The original contributions presented in the study are included in the article/Supplementary material, further inquiries can be directed to the corresponding author.
