# Supplementary material for: Public preferences for health data sharing governance: a discrete choice experiment in China
Source: Front Public Health. 2026 Oct 20;14:1883099. doi: 10.3389/fpubh.2026.1883099 (PMC13727597; doi:10.3389/fpubh.2026.1883099)
Supplement: Supplementary file 1 [file Data_Sheet_1.docx]

**Supplementary Material**

Public Preferences for Health Data Sharing Governance:
A Discrete Choice Experiment in China

This supplementary material reports design diagnostics, measurement validation, complete model estimates, and the supplementary analyses summarised in Section 4.5 of the main text, namely the robustness checks (Tables S21–S24), the penalized latent class analysis (Tables S14–S15, Figure S2) and the subgroup heterogeneity analysis (Tables S16–S17, Figure S3). Tables and figures are numbered with an S prefix.

**S1. DCE Design Diagnostics and Descriptive Statistics**

**Table S1.** Distribution of DCE attribute levels across all choice profiles

| **Attribute** | **Level** | **n** | **Proportion(%)** |
| --- | --- | --- | --- |
| Data Custodian | Public Hospital | 3577 | 48.3% |
| Data Custodian | Govt. Neutral Third Party | 3828 | 51.7% |
| Data Use Purpose | Medical Research | 2449 | 33.1% |
| Data Use Purpose | Epidemic Control | 2739 | 37.0% |
| Data Use Purpose | Personal Diagnosis | 2217 | 29.9% |
| Rights Protection | Basic Protection | 2310 | 31.2% |
| Rights Protection | Moderate Protection | 2186 | 29.5% |
| Rights Protection | Strong Protection | 2909 | 39.3% |
| Incentive | No Incentive | 1941 | 26.2% |
| Incentive | Health Points | 2205 | 29.8% |
| Incentive | Cash CNY 50 | 2090 | 28.2% |
| Incentive | Honorary Certificate | 1169 | 15.8% |
| Data Type | General Health Info | 5043 | 68.1% |
| Data Type | Sensitive Health Info | 2362 | 31.9% |

**Table S2.** Distribution of DCE attribute levels by questionnaire block

| **Block** | **Attribute** | **Level** | **n** | **Proportion(%)** |
| --- | --- | --- | --- | --- |
| Block1 | Data Custodian | Public Hospital | 1485 | 59.6% |
| Block1 | Data Custodian | Govt. Neutral Third Party | 1005 | 40.4% |
| Block1 | Data Use Purpose | Medical Research | 851 | 34.2% |
| Block1 | Data Use Purpose | Epidemic Control | 150 | 6.0% |
| Block1 | Data Use Purpose | Personal Diagnosis | 1489 | 59.8% |
| Block1 | Rights Protection | Basic Protection | 695 | 27.9% |
| Block1 | Rights Protection | Moderate Protection | 1021 | 41.0% |
| Block1 | Rights Protection | Strong Protection | 774 | 31.1% |
| Block1 | Incentive | No Incentive | 372 | 14.9% |
| Block1 | Incentive | Health Points | 968 | 38.9% |
| Block1 | Incentive | Cash CNY 50 | 479 | 19.2% |
| Block1 | Incentive | Honorary Certificate | 671 | 26.9% |
| Block1 | Data Type | General Health Info | 1639 | 65.8% |
| Block1 | Data Type | Sensitive Health Info | 851 | 34.2% |
| Block2 | Data Custodian | Public Hospital | 928 | 38.1% |
| Block2 | Data Custodian | Govt. Neutral Third Party | 1507 | 61.9% |
| Block2 | Data Use Purpose | Medical Research | 871 | 35.8% |
| Block2 | Data Use Purpose | Epidemic Control | 1504 | 61.8% |
| Block2 | Data Use Purpose | Personal Diagnosis | 60 | 2.5% |
| Block2 | Rights Protection | Basic Protection | 564 | 23.2% |
| Block2 | Rights Protection | Moderate Protection | 495 | 20.3% |
| Block2 | Rights Protection | Strong Protection | 1376 | 56.5% |
| Block2 | Incentive | No Incentive | 495 | 20.3% |
| Block2 | Incentive | Health Points | 661 | 27.1% |
| Block2 | Incentive | Cash CNY 50 | 1070 | 43.9% |
| Block2 | Incentive | Honorary Certificate | 209 | 8.6% |
| Block2 | Data Type | General Health Info | 1626 | 66.8% |
| Block2 | Data Type | Sensitive Health Info | 809 | 33.2% |
| Block3 | Data Custodian | Public Hospital | 1164 | 46.9% |
| Block3 | Data Custodian | Govt. Neutral Third Party | 1316 | 53.1% |
| Block3 | Data Use Purpose | Medical Research | 727 | 29.3% |
| Block3 | Data Use Purpose | Epidemic Control | 1085 | 43.8% |
| Block3 | Data Use Purpose | Personal Diagnosis | 668 | 26.9% |
| Block3 | Rights Protection | Basic Protection | 1051 | 42.4% |
| Block3 | Rights Protection | Moderate Protection | 670 | 27.0% |
| Block3 | Rights Protection | Strong Protection | 759 | 30.6% |
| Block3 | Incentive | No Incentive | 1074 | 43.3% |
| Block3 | Incentive | Health Points | 576 | 23.2% |
| Block3 | Incentive | Cash CNY 50 | 541 | 21.8% |
| Block3 | Incentive | Honorary Certificate | 289 | 11.7% |
| Block3 | Data Type | General Health Info | 1778 | 71.7% |
| Block3 | Data Type | Sensitive Health Info | 702 | 28.3% |

**Table S3.** Choice distribution across DCE tasks

| **Block** | **Task** | **n** | **Option A** | **Option A%** | **Option B** | **Option B%** |
| --- | --- | --- | --- | --- | --- | --- |
| Block1 | Task1 (Set #1) | 498 | 150 | 30.1% | 348 | 69.9% |
| Block1 | Task2 (Set #2) | 498 | 367 | 73.7% | 131 | 26.3% |
| Block1 | Task3 (Set #3) | 498 | 278 | 55.8% | 220 | 44.2% |
| Block1 | Task4 (Set #4) | 498 | 344 | 69.1% | 154 | 30.9% |
| Block1 | Task5 (Set #5) | 498 | 152 | 30.5% | 346 | 69.5% |
| Block2 | Task1 (Set #6) | 487 | 427 | 87.7% | 60 | 12.3% |
| Block2 | Task2 (Set #7) | 487 | 78 | 16.0% | 409 | 84.0% |
| Block2 | Task3 (Set #8) | 487 | 348 | 71.5% | 139 | 28.5% |
| Block2 | Task4 (Set #9) | 487 | 131 | 26.9% | 356 | 73.1% |
| Block2 | Task5 (Set #10) | 487 | 192 | 39.4% | 295 | 60.6% |
| Block3 | Task1 (Set #11) | 496 | 302 | 60.9% | 194 | 39.1% |
| Block3 | Task2 (Set #12) | 496 | 380 | 76.6% | 116 | 23.4% |
| Block3 | Task3 (Set #13) | 496 | 460 | 92.7% | 36 | 7.3% |
| Block3 | Task4 (Set #14) | 496 | 289 | 58.3% | 207 | 41.7% |
| Block3 | Task5 (Set #15) | 496 | 149 | 30.0% | 347 | 70.0% |

**Table S4.** Full demographic profile of respondents

| **Variable** | **Category** | **n** | **%** |
| --- | --- | --- | --- |
| Gender | Female | 943 | 63.7% |
|  | Male | 538 | 36.3% |
| Age | 18 years | 4 | 0.3% |
|  | 19-25 years | 482 | 32.5% |
|  | 26-35 years | 608 | 41.1% |
|  | 36-45 years | 260 | 17.6% |
|  | 46-55 years | 76 | 5.1% |
|  | ≥56 years | 51 | 3.4% |
| Residence | Urban | 1366 | 92.2% |
|  | Rural | 115 | 7.8% |
| Education | Middle school or below | 6 | 0.4% |
|  | High school/vocational | 34 | 2.3% |
|  | Junior college | 94 | 6.3% |
|  | Bachelor's | 1054 | 71.2% |
|  | Master's or above | 293 | 19.8% |
| Monthly Income | < CNY 3,000 | 214 | 14.4% |
|  | CNY 3,001-6,000 | 316 | 21.3% |
|  | CNY 6,001-10,000 | 479 | 32.3% |
|  | CNY 10,001-15,000 | 276 | 18.6% |
|  | > CNY 15,000 | 196 | 13.2% |
| Data-Related Job | Not at all | 326 | 22.0% |
|  | Slightly | 514 | 34.7% |
|  | Moderate | 344 | 23.2% |
|  | Considerably | 268 | 18.1% |
|  | Very closely | 29 | 2.0% |
| Hospital Visits (6 mo) | 0 times | 135 | 9.1% |
|  | 1-3 times | 1097 | 74.1% |
|  | 4-6 times | 198 | 13.4% |
|  | ≥7 times | 51 | 3.4% |

**Table S5.** Measurement items and descriptive statistics

| **Item** | **Scale/Dimension** | **Item Content** | **M** | **SD** |
| --- | --- | --- | --- | --- |
| T1 | Benevolence | Gov. protects citizens' data rights | 5.747 | 0.907 |
| T2 | Benevolence | Gov. policies serve public interest | 6.034 | 0.9 |
| T3 | Benevolence | Gov. sincerely aims to improve healthcare | 6.053 | 0.881 |
| T4 | Competence | Gov. is capable of managing health data | 5.795 | 0.944 |
| T5 | Competence | Gov. is professional in data management | 5.564 | 1.028 |
| T6 | Competence | Gov. performs well in data management duties | 5.517 | 0.983 |
| T7 | Integrity | Gov. is transparent in explaining data policy | 5.512 | 1.06 |
| T8 | Integrity | Gov. is trustworthy in handling health data | 5.766 | 0.97 |
| T9 | Integrity | Gov. is honest in data management | 5.82 | 0.956 |
| (Mean) | Benevolence (T1-T3) | Dimension mean | 5.945 | 0.69 |
| (Mean) | Competence (T4-T6) | Dimension mean | 5.625 | 0.776 |
| (Mean) | Integrity (T7-T9) | Dimension mean | 5.699 | 0.831 |
| (Mean) | Gov. Org. Trust (T1-T9) | Overall mean | 5.756 | 0.675 |
| W1 | Sharing Willingness | Willing to share medical data overall | 5.386 | 0.924 |
| W2 | Sharing Willingness | Willing to allow authorized use of health info | 5.492 | 1.015 |
| W3 | Sharing Willingness | Would provide data if organization requests | 5.483 | 1.027 |
| W4 | Sharing Willingness | Would consider participating in data sharing | 5.714 | 0.987 |
| (Mean) | Sharing Willingness (W1-W4) | Overall mean | 5.518 | 0.799 |

**S2. Measurement Validation**

**Table S6.** Confirmatory factor analysis model fit

| **Model** | **^2** | **df** | **^2/df** | **CFI** | **TLI** | **RMSEA** | **Evaluation** |
| --- | --- | --- | --- | --- | --- | --- | --- |
| 1F: Single-factor (Trust) | 180.666 | 27 | 6.691 | 0.969 | 0.958 | 0.062 | CFI excellent, TLI excellent, RMSEA acceptable |
| 2F (selected): Two-factor (Trust+Willingness) | 258.567 | 64 | 4.04 | 0.974 | 0.968 | 0.045 | CFI excellent, TLI excellent, RMSEA excellent |
| 3F: Three-factor (Ben./Comp./Int.) | 144.293 | 24 | 6.012 | 0.976 | 0.963 | 0.058 | CFI excellent, TLI excellent, RMSEA excellent |
| 4F: Four-factor (3-dim Trust+Will.) | 208.895 | 59 | 3.541 | 0.98 | 0.974 | 0.041 | CFI excellent, TLI excellent, RMSEA excellent |

**Table S7.** Standardized factor loadings

| **Item** | **Factor** | **Std. Loading (λ)** | **Significance** |
| --- | --- | --- | --- |
| T1 | Gov. Org. Trust | 0.743 | Fixed |
| T2 | Gov. Org. Trust | 0.553 | *** |
| T3 | Gov. Org. Trust | 0.559 | *** |
| T4 | Gov. Org. Trust | 0.61 | *** |
| T5 | Gov. Org. Trust | 0.577 | *** |
| T6 | Gov. Org. Trust | 0.698 | *** |
| T7 | Gov. Org. Trust | 0.661 | *** |
| T8 | Gov. Org. Trust | 0.736 | *** |
| T9 | Gov. Org. Trust | 0.783 | *** |
| W1 | Sharing Willingness | 0.806 | Fixed |
| W2 | Sharing Willingness | 0.732 | *** |
| W3 | Sharing Willingness | 0.672 | *** |
| W4 | Sharing Willingness | 0.732 | *** |

**Table S8.** Convergent reliability and internal consistency

| **Construct** | **N Items** | **AVE** | **AVE** | **CR** | **Cronbach's** | **Evaluation** |
| --- | --- | --- | --- | --- | --- | --- |
| Gov. Org. Trust | 9 | 0.439 | 0.663 | 0.874 | 0.872 | AVE0.5 No, CR>0.7 Yes |
| Sharing Willingness | 4 | 0.543 | 0.737 | 0.826 | 0.822 | AVE>0.5 Yes, CR>0.7 Yes |

**Table S9.** Discriminant validity based on HTMT ratios

| **Construct Pair** | **HTMT** | **Bootstrap 95% CI** | **CI Upper<1.0** | **Judgment(<0.85)** |
| --- | --- | --- | --- | --- |
| Gov. Org. Trust vs Sharing Willingness | 0.629 | [0.557, 0.697] | Yes | Yes |
| Benevolence vs Competence | 0.901 | [0.853, 0.946] | Yes | No |
| Benevolence vs Integrity | 0.998 | [0.962, 1.038] | No | No |
| Competence vs Integrity | 0.901 | [0.86, 0.939] | Yes | No |
| Benevolence vs Willingness | 0.6 | [0.518, 0.681] | Yes | Yes |
| Competence vs Willingness | 0.652 | [0.578, 0.724] | Yes | Yes |
| Integrity vs Willingness | 0.59 | [0.513, 0.663] | Yes | Yes |

**S3. Main and Conditional AMCE Estimates**

**Table S10.** Main AMCE estimates for institutional attributes

| **Attribute** | **Level** | **AMCE** | **SE** | **95%CI Lower** | **95%CI Upper** | **z** | **p** | **Sig.** |
| --- | --- | --- | --- | --- | --- | --- | --- | --- |
| Data Custodian | Public Hospital (Ref.) | - | - | - | - | - | - | Reference |
| Data Custodian | Govt. Neutral Third Party | 0.0594 | 0.0119 | 0.0361 | 0.0827 | 4.994 | 0.0 | *** |
| Data Use Purpose | Medical Research (Ref.) | - | - | - | - | - | - | Reference |
| Data Use Purpose | Epidemic Control | 0.0082 | 0.0105 | -0.0123 | 0.0287 | 0.782 | 0.4341 | ns |
| Data Use Purpose | Personal Diagnosis | -0.103 | 0.011 | -0.1245 | -0.0815 | -9.407 | 0.0 | *** |
| Rights Protection | Basic Protection (Ref.) | - | - | - | - | - | - | Reference |
| Rights Protection | Moderate Protection | 0.003 | 0.0121 | -0.0207 | 0.0268 | 0.249 | 0.8032 | ns |
| Rights Protection | Strong Protection | 0.0779 | 0.012 | 0.0544 | 0.1015 | 6.493 | 0.0 | *** |
| Incentive | No Incentive (Ref.) | - | - | - | - | - | - | Reference |
| Incentive | Health Points | 0.1292 | 0.0128 | 0.104 | 0.1543 | 10.075 | 0.0 | *** |
| Incentive | Cash CNY 50 | 0.1447 | 0.0117 | 0.1218 | 0.1677 | 12.378 | 0.0 | *** |
| Incentive | Honorary Certificate | -0.0155 | 0.0123 | -0.0395 | 0.0086 | -1.259 | 0.2082 | ns |
| Data Type | Sensitive Health Info (Ref.) | - | - | - | - | - | - | Reference |
| Data Type | General Health Info | 0.3205 | 0.0111 | 0.2988 | 0.3422 | 28.905 | 0.0 | *** |

**Table S11.** Conditional AMCEs by data type and interaction estimates

| **Attribute** | **Level** | **Reference** | **AMCE_Sensitive** | **95%CI_sens** | **sig_sens** | **AMCE_General** | **95%CI_gen** | **sig_gen** | **Interaction** | **95%CI_int** | **p_BH** | **sig_BH** |
| --- | --- | --- | --- | --- | --- | --- | --- | --- | --- | --- | --- | --- |
| Data Custodian | Govt. Third Party | vs Public Hospital | -0.0478 | [-0.0742, -0.0214] | *** | 0.0803 | [0.0548, 0.1059] | *** | 0.1281 | [0.108, 0.1482] | 0.0 | *** |
| Data Use Purpose | Epidemic Control | vs Medical Research | -0.0422 | [-0.0684, -0.0159] | ** | -0.0027 | [-0.031, 0.0256] | ns | 0.0395 | [0.0122, 0.0668] | 0.0045 | ** |
| Data Use Purpose | Personal Diagnosis | vs Medical Research | -0.3333 | [-0.3664, -0.3002] | *** | -0.1052 | [-0.1329, -0.0775] | *** | 0.2281 | [0.1989, 0.2574] | 0.0 | *** |
| Rights Protection | Moderate Protection | vs Basic Protection | 0.037 | [0.0053, 0.0686] | * | -0.0009 | [-0.0335, 0.0317] | ns | -0.0379 | [-0.0637, -0.0121] | 0.0045 | ** |
| Rights Protection | Strong Protection | vs Basic Protection | 0.2208 | [0.1936, 0.2479] | *** | -0.0212 | [-0.0488, 0.0063] | ns | -0.242 | [-0.271, -0.213] | 0.0 | *** |
| Incentive | Health Points | vs No Incentive | -0.0296 | [-0.0573, -0.0019] | * | 0.2436 | [0.2122, 0.275] | *** | 0.2732 | [0.2426, 0.3038] | 0.0 | *** |
| Incentive | Cash CNY 50 | vs No Incentive | 0.0756 | [0.0433, 0.1079] | *** | 0.1794 | [0.152, 0.2067] | *** | 0.1038 | [0.0667, 0.1408] | 0.0 | *** |
| Incentive | Honorary Certificate | vs No Incentive | -0.1096 | [-0.141, -0.0781] | *** | 0.0578 | [0.024, 0.0915] | *** | 0.1673 | [0.1308, 0.2039] | 0.0 | *** |

**Table S12.** Moderation of attribute preferences by government organizational trust

| **Attribute** | **Level** | **Reference** | **β(Trust×Attr)** | **SE** | **95%CI** | **p_raw** | **sig_raw** | **p_BH** | **sig_BH** |
| --- | --- | --- | --- | --- | --- | --- | --- | --- | --- |
| Data Custodian | Govt. Third Party | vs Public Hospital | 0.0511 | 0.0175 | [0.0169,0.0853] | 0.0034 | ** | 0.0302 | * |
| Data Use Purpose | Epidemic Control | vs Medical Research | -0.0049 | 0.0155 | [-0.0352,0.0254] | 0.752 | ns | 0.752 | ns |
| Data Use Purpose | Personal Diagnosis | vs Medical Research | -0.0132 | 0.0156 | [-0.0438,0.0174] | 0.3989 | ns | 0.5984 | ns |
| Rights Protection | Moderate Protection | vs Basic Protection | 0.01 | 0.0164 | [-0.0221,0.042] | 0.5429 | ns | 0.698 | ns |
| Rights Protection | Strong Protection | vs Basic Protection | -0.0165 | 0.0174 | [-0.0507,0.0177] | 0.3442 | ns | 0.5984 | ns |
| Incentive | Health Points | vs No Incentive | -0.052 | 0.0192 | [-0.0896,-0.0144] | 0.0067 | ** | 0.0302 | * |
| Incentive | Cash CNY 50 | vs No Incentive | -0.0232 | 0.0186 | [-0.0597,0.0134] | 0.2145 | ns | 0.4826 | ns |
| Incentive | Honorary Certificate | vs No Incentive | -0.0062 | 0.0184 | [-0.0422,0.0298] | 0.7356 | ns | 0.752 | ns |
| Data Type | General Health Info | vs Sensitive Health Info | -0.0351 | 0.0147 | [-0.064,-0.0062] | 0.0173 | * | 0.0519 | † |

**Table S13.** Group-specific AMCEs by government organizational trust

| **Attribute** | **Level** | **Reference** | **Higher-Trust AMCE** | **SE_H** | **95%CI_H** | **sig_H** | **Lower-Trust AMCE** | **SE_L** | **95%CI_L** | **sig_L** |
| --- | --- | --- | --- | --- | --- | --- | --- | --- | --- | --- |
| Data Custodian | Govt. Third Party | vs Public Hospital | 0.0691 | 0.0165 | [0.0368,0.1014] | *** | 0.0473 | 0.0171 | [0.0137,0.0809] | ** |
| Data Use Purpose | Epidemic Control | vs Medical Research | -0.0006 | 0.014 | [-0.0282,0.0269] | ns | 0.0193 | 0.0157 | [-0.0114,0.05] | ns |
| Data Use Purpose | Personal Diagnosis | vs Medical Research | -0.1179 | 0.0155 | [-0.1483,-0.0876] | *** | -0.0842 | 0.0153 | [-0.1143,-0.0542] | *** |
| Rights Protection | Moderate Protection | vs Basic Protection | 0.0128 | 0.0168 | [-0.0202,0.0458] | ns | -0.0088 | 0.0174 | [-0.0428,0.0252] | ns |
| Rights Protection | Strong Protection | vs Basic Protection | 0.0758 | 0.0162 | [0.0442,0.1075] | *** | 0.0808 | 0.018 | [0.0455,0.116] | *** |
| Incentive | Health Points | vs No Incentive | 0.1101 | 0.0172 | [0.0765,0.1438] | *** | 0.1526 | 0.0192 | [0.115,0.1903] | *** |
| Incentive | Cash CNY 50 | vs No Incentive | 0.1406 | 0.0156 | [0.1101,0.1711] | *** | 0.1496 | 0.0177 | [0.1149,0.1843] | *** |
| Incentive | Honorary Certificate | vs No Incentive | -0.0096 | 0.0167 | [-0.0423,0.0231] | ns | -0.0223 | 0.0181 | [-0.0578,0.0132] | ns |
| Data Type | General Health Info | vs Sensitive Health Info | 0.2903 | 0.0155 | [0.2599,0.3208] | *** | 0.3571 | 0.0157 | [0.3264,0.3878] | *** |

Note: Groups were defined by a median split of the government organizational trust score (median = 5.889; higher-trust group n = 809, mean = 6.19; lower-trust group n = 672, mean = 5.23). The lower-trust group also scored above the scale midpoint, so the labels denote relative levels of trust. Inference on moderation is based on the continuous interaction model (Table S12).

**S4. Latent Class and Heterogeneity Analyses**

**Table S14.** Latent class model fit comparison (penalized maximum-likelihood, L_2 penalty λ=1). BIC(occ) and BIC(resp) use choice occasions (N=7405) and respondents (N=1481) as the sample-size term in the BIC penalty and both marginally favor the four-class model; that solution, however, was recovered in only 2 of 50 random starts, whereas the three-class solution was recovered in 9 of 50. Stability = random starts reaching the best solution. The three-class solution is reported as the most stable interpretable summary; the class count is not treated as definitive

| **Model** | **n_params** | **LL** | **BIC(occ)** | **BIC(resp)** | **Stability** | **Norm. entropy↓** | **Class. quality↑** |
| --- | --- | --- | --- | --- | --- | --- | --- |
| Q=1 | 9 | -4499.32 | 9078.83 | 9064.34 | 5/5 | - | - |
| Q=2 | 19 | -4188.96 | 8547.21 | 8516.63 | 17/50 | 0.296 | 0.704 |
| Q=3 [reported] | 29 | -4084.17 | 8426.72 | 8380.05 | 9/50 | 0.316 | 0.684 |
| Q=4 [BIC-occ min] [BIC-resp min] | 39 | -4031.93 | 8411.36 | 8348.59 | 2/50 | 0.312 | 0.688 |

**Table S15.** Class-specific preference estimates for the three-class solution (penalized maximum-likelihood, L_2 penalty λ=1; classes ordered by size). Estimation is penalized because the unpenalized latent class logit likelihood does not attain a finite maximum in this design (five choice tasks per respondent, nine class-specific parameters). Standard errors are conditional on estimated class memberships and do not incorporate classification uncertainty; average posterior assignment probabilities were 0.891, 0.862, and 0.845. Class labels are descriptive operational labels based on the sign and relative magnitude of the coefficients, not validated population segments

| **Attribute** | **Class 1 β** | **Class 2 β** | **Class 3 β** | **Class 1 SE** | **Class 2 SE** | **Class 3 SE** | **Class 1 sig** | **Class 2 sig** | **Class 3 sig** |
| --- | --- | --- | --- | --- | --- | --- | --- | --- | --- |
| Class Size (π) | 0.197 | 0.290 | 0.513 |  |  |  |  |  |  |
| Govt. Third Party vs Public Hospital | -0.095 | -1.8973 | 0.6123 | 0.0544 | 0.0614 | 0.0459 | † | *** | *** |
| Epidemic Control vs Medical Research | 0.3866 | -1.9128 | 0.3201 | 0.0659 | 0.104 | 0.0555 | *** | *** | *** |
| Personal Diagnosis vs Medical Research | 0.0001 | -3.3206 | 0.7835 | 0.0668 | 0.0649 | 0.0594 | ns | *** | *** |
| Moderate Protection vs Basic Protection | 0.121 | 1.9986 | -1.2304 | 0.0712 | 0.0763 | 0.0558 | † | *** | *** |
| Strong Protection vs Basic Protection | 0.073 | -1.4925 | 1.3283 | 0.0638 | 0.0751 | 0.0521 | ns | *** | *** |
| Health Points vs No Incentive | 0.4683 | -0.0974 | 2.7648 | 0.0744 | 0.0733 | 0.0782 | *** | ns | *** |
| Cash ¥50 vs No Incentive | 0.6541 | 0.6947 | 1.4919 | 0.0795 | 0.1015 | 0.0542 | *** | *** | *** |
| Honorary Cert. vs No Incentive | 0.216 | -0.897 | 0.2319 | 0.0845 | 0.0884 | 0.0793 | * | *** | ** |
| General Health Info vs Sensitive Info | -0.141 | 1.9806 | 2.8013 | 0.0563 | 0.0645 | 0.0486 | * | *** | *** |

**Table S16.** Demographic heterogeneity in attribute effects

| **Dimension** | **Attribute** | **Group1** | **Group1_AMCE** | **95%CI_1** | **sig1** | **Group0** | **Group0_AMCE** | **95%CI_0** | **sig0** | **Difference** | **p_BH** | **sig_BH** |
| --- | --- | --- | --- | --- | --- | --- | --- | --- | --- | --- | --- | --- |
| Gender | Govt. Third Party vs Pub. Hospital | Male | 0.084 | [0.0459, 0.1222] | *** | Female | 0.0452 | [0.0158, 0.0747] | ** | 0.0388 | 0.2575 | ns |
| Gender | Epidemic Control vs Med. Research | Male | 0.0101 | [-0.0222, 0.0424] | ns | Female | 0.0085 | [-0.0178, 0.0348] | ns | 0.0016 | 0.9409 | ns |
| Gender | Personal Diag. vs Med. Research | Male | -0.1041 | [-0.14, -0.0683] | *** | Female | -0.1019 | [-0.1287, -0.075] | *** | -0.0023 | 0.9409 | ns |
| Gender | Moderate Prot. vs Basic Prot. | Male | 0.0057 | [-0.0346, 0.046] | ns | Female | 0.0033 | [-0.026, 0.0325] | ns | 0.0024 | 0.9409 | ns |
| Gender | Strong Prot. vs Basic Prot. | Male | 0.0499 | [0.0117, 0.0881] | * | Female | 0.0942 | [0.0644, 0.124] | *** | -0.0443 | 0.2198 | ns |
| Gender | Health Points vs No Incentive | Male | 0.153 | [0.1095, 0.1966] | *** | Female | 0.1173 | [0.0865, 0.148] | *** | 0.0358 | 0.3383 | ns |
| Gender | Cash CNY 50 vs No Incentive | Male | 0.184 | [0.1453, 0.2226] | *** | Female | 0.1225 | [0.0941, 0.1509] | *** | 0.0614 | 0.0542 | † |
| Gender | Honorary Cert. vs No Incentive | Male | -0.0195 | [-0.0607, 0.0217] | ns | Female | -0.0125 | [-0.0419, 0.017] | ns | -0.007 | 0.9409 | ns |
| Gender | General Info vs Sensitive Info | Male | 0.2806 | [0.2441, 0.3171] | *** | Female | 0.3434 | [0.3164, 0.3704] | *** | -0.0628 | 0.0542 | † |
| Age | Govt. Third Party vs Pub. Hospital | 35 yrs | 0.05 | [0.0228, 0.0773] | *** | ≥36 yrs | 0.0837 | [0.0385, 0.1289] | *** | -0.0337 | 0.5293 | ns |
| Age | Epidemic Control vs Med. Research | 35 yrs | 0.0181 | [-0.006, 0.0423] | ns | ≥36 yrs | -0.0188 | [-0.0573, 0.0198] | ns | 0.0369 | 0.5293 | ns |
| Age | Personal Diag. vs Med. Research | 35 yrs | -0.0966 | [-0.1214, -0.0718] | *** | ≥36 yrs | -0.1212 | [-0.1642, -0.0781] | *** | 0.0246 | 0.5293 | ns |
| Age | Moderate Prot. vs Basic Prot. | 35 yrs | -0.0028 | [-0.0305, 0.0248] | ns | ≥36 yrs | 0.0198 | [-0.0268, 0.0664] | ns | -0.0227 | 0.5293 | ns |
| Age | Strong Prot. vs Basic Prot. | 35 yrs | 0.0849 | [0.0572, 0.1126] | *** | ≥36 yrs | 0.0605 | [0.0157, 0.1054] | ** | 0.0244 | 0.5293 | ns |
| Age | Health Points vs No Incentive | 35 yrs | 0.1389 | [0.1092, 0.1687] | *** | ≥36 yrs | 0.1037 | [0.0571, 0.1504] | *** | 0.0352 | 0.5293 | ns |
| Age | Cash CNY 50 vs No Incentive | 35 yrs | 0.1477 | [0.1205, 0.1749] | *** | ≥36 yrs | 0.1356 | [0.0931, 0.178] | *** | 0.0121 | 0.7165 | ns |
| Age | Honorary Cert. vs No Incentive | 35 yrs | -0.0065 | [-0.0346, 0.0216] | ns | ≥36 yrs | -0.0388 | [-0.0856, 0.0079] | ns | 0.0323 | 0.5293 | ns |
| Age | General Info vs Sensitive Info | 35 yrs | 0.3234 | [0.2983, 0.3484] | *** | ≥36 yrs | 0.3141 | [0.2703, 0.358] | *** | 0.0092 | 0.7204 | ns |
| Education | Govt. Third Party vs Pub. Hospital | Bachelor+ | 0.0587 | [0.0342, 0.0833] | *** | Below Bach. | 0.0641 | [-0.0108, 0.1389] | † | -0.0053 | 0.9554 | ns |
| Education | Epidemic Control vs Med. Research | Bachelor+ | 0.009 | [-0.0124, 0.0304] | ns | Below Bach. | 0.0068 | [-0.0659, 0.0795] | ns | 0.0022 | 0.9554 | ns |
| Education | Personal Diag. vs Med. Research | Bachelor+ | -0.1074 | [-0.1301, -0.0848] | *** | Below Bach. | -0.0574 | [-0.1248, 0.0099] | † | -0.05 | 0.5565 | ns |
| Education | Moderate Prot. vs Basic Prot. | Bachelor+ | 0.0064 | [-0.0187, 0.0315] | ns | Below Bach. | -0.0244 | [-0.0987, 0.0499] | ns | 0.0308 | 0.6588 | ns |
| Education | Strong Prot. vs Basic Prot. | Bachelor+ | 0.0769 | [0.0522, 0.1015] | *** | Below Bach. | 0.0908 | [0.0117, 0.1699] | * | -0.0139 | 0.9525 | ns |
| Education | Health Points vs No Incentive | Bachelor+ | 0.1263 | [0.0998, 0.1528] | *** | Below Bach. | 0.1628 | [0.083, 0.2427] | *** | -0.0365 | 0.6588 | ns |
| Education | Cash CNY 50 vs No Incentive | Bachelor+ | 0.1395 | [0.1154, 0.1636] | *** | Below Bach. | 0.1966 | [0.1214, 0.2718] | *** | -0.0571 | 0.5565 | ns |
| Education | Honorary Cert. vs No Incentive | Bachelor+ | -0.0211 | [-0.0462, 0.0039] | † | Below Bach. | 0.0396 | [-0.0472, 0.1264] | ns | -0.0607 | 0.5565 | ns |
| Education | General Info vs Sensitive Info | Bachelor+ | 0.3174 | [0.2944, 0.3404] | *** | Below Bach. | 0.3514 | [0.2843, 0.4184] | *** | -0.0339 | 0.6588 | ns |
| Gov. Trust | Govt. Third Party vs Pub. Hospital | Higher Trust | 0.0691 | [0.0368, 0.1014] | *** | Lower Trust | 0.0473 | [0.0137, 0.0809] | ** | 0.0218 | 0.557 | ns |
| Gov. Trust | Epidemic Control vs Med. Research | Higher Trust | -0.0006 | [-0.0282, 0.0269] | ns | Lower Trust | 0.0193 | [-0.0114, 0.05] | ns | -0.0199 | 0.557 | ns |
| Gov. Trust | Personal Diag. vs Med. Research | Higher Trust | -0.1179 | [-0.1483, -0.0876] | *** | Lower Trust | -0.0842 | [-0.1143, -0.0542] | *** | -0.0337 | 0.3662 | ns |
| Gov. Trust | Moderate Prot. vs Basic Prot. | Higher Trust | 0.0128 | [-0.0202, 0.0458] | ns | Lower Trust | -0.0088 | [-0.0428, 0.0252] | ns | 0.0216 | 0.557 | ns |
| Gov. Trust | Strong Prot. vs Basic Prot. | Higher Trust | 0.0758 | [0.0442, 0.1075] | *** | Lower Trust | 0.0808 | [0.0455, 0.116] | *** | -0.0049 | 0.8378 | ns |
| Gov. Trust | Health Points vs No Incentive | Higher Trust | 0.1101 | [0.0765, 0.1438] | *** | Lower Trust | 0.1526 | [0.115, 0.1903] | *** | -0.0425 | 0.3662 | ns |
| Gov. Trust | Cash CNY 50 vs No Incentive | Higher Trust | 0.1406 | [0.1101, 0.1711] | *** | Lower Trust | 0.1496 | [0.1149, 0.1843] | *** | -0.009 | 0.7895 | ns |
| Gov. Trust | Honorary Cert. vs No Incentive | Higher Trust | -0.0096 | [-0.0423, 0.0231] | ns | Lower Trust | -0.0223 | [-0.0578, 0.0132] | ns | 0.0127 | 0.7784 | ns |
| Gov. Trust | General Info vs Sensitive Info | Higher Trust | 0.2903 | [0.2599, 0.3208] | *** | Lower Trust | 0.3571 | [0.3264, 0.3878] | *** | -0.0668 | 0.0223 | * |

**Table S17.** Key subgroup differences after BH correction

| **Group Variable** | **Attribute** | **Group1 AMCE** | **Group0 AMCE** | **Difference** | **SE** | **p_raw** | **p_BH** | **Sig.** |
| --- | --- | --- | --- | --- | --- | --- | --- | --- |
| Gov. Trust | General Info vs vs Sensitive Info | 0.2903 | 0.3571 | -0.0668 | 0.0221 | 0.0025 | 0.0223 | * |
| Gender | Cash CNY 50 vs vs No Incentive | 0.184 | 0.1225 | 0.0614 | 0.0245 | 0.012 | 0.0542 | † |
| Gender | General Info vs vs Sensitive Info | 0.2806 | 0.3434 | -0.0628 | 0.0232 | 0.0067 | 0.0542 | † |

**S5. Auxiliary Willingness Analyses**

**Table S18.** Association between sharing willingness and continuous trust score

| **Variable Pair** | **N** | **Pearson r** | **sig_Pearson** | **Spearman** | **sig_Spearman** |
| --- | --- | --- | --- | --- | --- |
| Gov. Org. Trust × Sharing Willingness | 1481 | 0.534 | *** | 0.555 | *** |
| Gov. Org. Trust × DCE Sensitive Choice Rate | 1481 | 0.071 | ** | 0.078 | ** |
| Sharing Willingness × DCE Sensitive Choice Rate | 1481 | 0.13 | *** | 0.152 | *** |

**Table S19.** OLS models for sharing willingness

| **Model** | **Variable** | **β** | **SE** | **95%CI** | **p** | **Sig.** | **Std.β** |
| --- | --- | --- | --- | --- | --- | --- | --- |
| Model 1 | Intercept | 1.882 | 0.1509 | [1.5861,2.1780] | 0.0 | *** | - |
| Model 1 | Gov. Org. Trust | 0.6317 | 0.026 | [0.5807,0.6828] | 0.0 | *** | 0.5337 |
| Model 1 | Model Statistics |  |  |  |  |  |  |
| Model 1 | R2=0.2848 | AdjR2=0.2843 | F=588.949 | p=0.0000 | N=1481 |  |  |
| Model 2 | Intercept | 1.6123 | 0.2119 | [1.1966,2.0280] | 0.0 | *** | - |
| Model 2 | Gov. Org. Trust | 0.6045 | 0.0262 | [0.5531,0.6558] | 0.0 | *** | 0.5106 |
| Model 2 | Gender (Male=1) | 0.0977 | 0.0366 | [0.0258,0.1696] | 0.0078 | ** | 0.0588 |
| Model 2 | Age (ordinal) | 0.0004 | 0.0196 | [-0.0380,0.0388] | 0.9845 | ns | 0.0005 |
| Model 2 | Residence (Urban=1) | 0.0853 | 0.0671 | [-0.0464,0.2170] | 0.2041 | ns | 0.0286 |
| Model 2 | Education (ordinal) | -0.0224 | 0.0299 | [-0.0812,0.0363] | 0.4537 | ns | -0.0174 |
| Model 2 | Monthly Income (ordinal) | 0.0538 | 0.0172 | [0.0201,0.0875] | 0.0018 | ** | 0.0827 |
| Model 2 | Data-Related Job | -0.0407 | 0.0162 | [-0.0726,-0.0088] | 0.0124 | * | -0.055 |
| Model 2 | Hospital Visits (6 mo) | 0.1625 | 0.0293 | [0.1050,0.2200] | 0.0 | *** | 0.1204 |
| Model 2 | Model Statistics |  |  |  |  |  |  |
| Model 2 | R2=0.3150 | AdjR2=0.3113 | F=84.607 | p=0.0000 | N=1481 |  |  |

**Table S20.** Sharing willingness by trust group

| **Group** | **N** | **M** | **SD** | **Welch t** | **p** | **Sig.** | **Cohen's d** |
| --- | --- | --- | --- | --- | --- | --- | --- |
| Higher Trust | 809 | 5.827 | 0.647 |  |  |  |  |
| Lower Trust | 672 | 5.147 | 0.806 | 17.672 | 0.0 | *** | 0.941 |

**S6. Robustness Checks**

**Table S21.** Robustness check comparing OLS and conditional logit specifications

| **Attribute** | **OLS-β** | **OLS-SE** | **OLS-sig** | **Clogit-β** | **Clogit-SE** | **Clogit-sig** | **Clogit-AME(approx.)** |
| --- | --- | --- | --- | --- | --- | --- | --- |
| Govt. Third Party | 0.0594 | 0.0119 | *** | 0.1791 | 0.0298 | *** | 0.0376 |
| Epidemic Control | 0.0082 | 0.0105 | ns | 0.0909 | 0.0321 | ** | 0.0191 |
| Personal Diagnosis | -0.103 | 0.011 | *** | -0.2994 | 0.0359 | *** | -0.0629 |
| Moderate Protection | 0.003 | 0.0121 | ns | 0.0669 | 0.0384 | † | 0.014 |
| Strong Protection | 0.0779 | 0.012 | *** | 0.4039 | 0.0419 | *** | 0.0849 |
| Health Points | 0.1292 | 0.0128 | *** | 0.3775 | 0.041 | *** | 0.0793 |
| Cash CNY 50 | 0.1447 | 0.0117 | *** | 0.5156 | 0.0388 | *** | 0.1083 |
| Honorary Cert. | -0.0155 | 0.0123 | ns | -0.0489 | 0.0421 | ns | -0.0103 |
| General Health Info | 0.3205 | 0.0111 | *** | 0.8068 | 0.0319 | *** | 0.1695 |

Note: The conditional logit was estimated on the differenced attributes of the two alternatives without an intercept, with respondent-clustered standard errors. Signs agree for all nine attribute levels; epidemic control and moderate protection reach significance only in the conditional logit. Clogit-AME (approx.) is the coefficient scaled by the mean of p(1 − p).

**Table S22.** Robustness check using alternative response-time threshold

| **Attribute** | **Main β(≥100s)** | **SE** | **sig** | **Sensitive β(>160s)** | **SE** | **sig** | **Diff** | **Sign Change** |
| --- | --- | --- | --- | --- | --- | --- | --- | --- |
| Govt. Third Party | 0.0594 | 0.0119 | *** | 0.0589 | 0.0121 | *** | -0.0005 | NO |
| Epidemic Control | 0.0082 | 0.0105 | ns | 0.0081 | 0.0106 | ns | -0.0001 | NO |
| Personal Diagnosis | -0.103 | 0.011 | *** | -0.1022 | 0.0111 | *** | 0.0008 | NO |
| Moderate Protection | 0.003 | 0.0121 | ns | 0.0037 | 0.0122 | ns | 0.0007 | NO |
| Strong Protection | 0.0779 | 0.012 | *** | 0.0767 | 0.0121 | *** | -0.0012 | NO |
| Health Points | 0.1292 | 0.0128 | *** | 0.1305 | 0.013 | *** | 0.0013 | NO |
| Cash CNY 50 | 0.1447 | 0.0117 | *** | 0.1454 | 0.0118 | *** | 0.0007 | NO |
| Honorary Cert. | -0.0155 | 0.0123 | ns | -0.015 | 0.0124 | ns | 0.0005 | NO |
| General Health Info | 0.3205 | 0.0111 | *** | 0.3192 | 0.0112 | *** | -0.0013 | NO |

**Table S23.** Robustness check excluding the highly imbalanced choice set (#13)

| **Attribute** | **Full Sample β** | **Exclude #13 β** | **Diff** | **Sign Change** | **Pass** |
| --- | --- | --- | --- | --- | --- |
| Govt. Third Party | 0.0594 | 0.0256 | -0.0338 | NO | YES |
| Epidemic Control | 0.0082 | -0.0179 | -0.0261 | YES | NO |
| Personal Diagnosis | -0.103 | -0.0759 | 0.0271 | NO | YES |
| Moderate Protection | 0.003 | 0.0176 | 0.0146 | NO | YES |
| Strong Protection | 0.0779 | 0.1214 | 0.0435 | NO | YES |
| Health Points | 0.1292 | 0.0566 | -0.0726 | NO | YES |
| Cash CNY 50 | 0.1447 | 0.1232 | -0.0215 | NO | YES |
| Honorary Cert. | -0.0155 | -0.0354 | -0.0199 | NO | YES |
| General Health Info | 0.3205 | 0.279 | -0.0415 | NO | YES |

**Table S24.** Robustness check including respondent covariates

| **Attribute** | **Main Effect β** | **Main sig** | **With Covars β** | **SE** | **95%CI** | **sig** | **Sign Change** |
| --- | --- | --- | --- | --- | --- | --- | --- |
| Govt. Third Party | 0.0594 | *** | 0.0594 | 0.0119 | [0.0361,0.0827] | *** | NO |
| Epidemic Control | 0.0082 | ns | 0.0081 | 0.0105 | [-0.0124,0.0287] | ns | NO |
| Personal Diagnosis | -0.103 | *** | -0.103 | 0.011 | [-0.1245,-0.0816] | *** | NO |
| Moderate Protection | 0.003 | ns | 0.003 | 0.0121 | [-0.0207,0.0268] | ns | NO |
| Strong Protection | 0.0779 | *** | 0.0779 | 0.012 | [0.0544,0.1015] | *** | NO |
| Health Points | 0.1292 | *** | 0.1292 | 0.0128 | [0.1041,0.1544] | *** | NO |
| Cash CNY 50 | 0.1447 | *** | 0.1448 | 0.0117 | [0.1219,0.1677] | *** | NO |
| Honorary Cert. | -0.0155 | ns | -0.0154 | 0.0123 | [-0.0395,0.0087] | ns | NO |
| General Health Info | 0.3205 | *** | 0.3205 | 0.0111 | [0.2988,0.3423] | *** | NO |

**S7. Supplementary Figures**

**Figure S1. Conditional AMCEs for higher- and lower-trust groups (median split of the trust score; median = 5.889). Points denote point estimates and bars show 95% confidence intervals; Δ marks attribute levels whose interaction with continuous trust was significant after BH correction.**

**Figure S2. Class-specific preference estimates in the penalized three-class latent class solution.**

**Figure S3. Subgroup heterogeneity in attribute effects by gender, age, education and trust group.**
