# Supplementary material for: Public preferences for health data sharing governance: a discrete choice experiment in China
Source: Front Public Health. 2026 Oct 20;14:1883099. doi: 10.3389/fpubh.2026.1883099 (PMC13727597; doi:10.3389/fpubh.2026.1883099)
Supplement: Supplementary file 2 [file Data_Sheet_2.ZIP › Reproducibility_Package/results/figures/amce_forest_en.pdf]

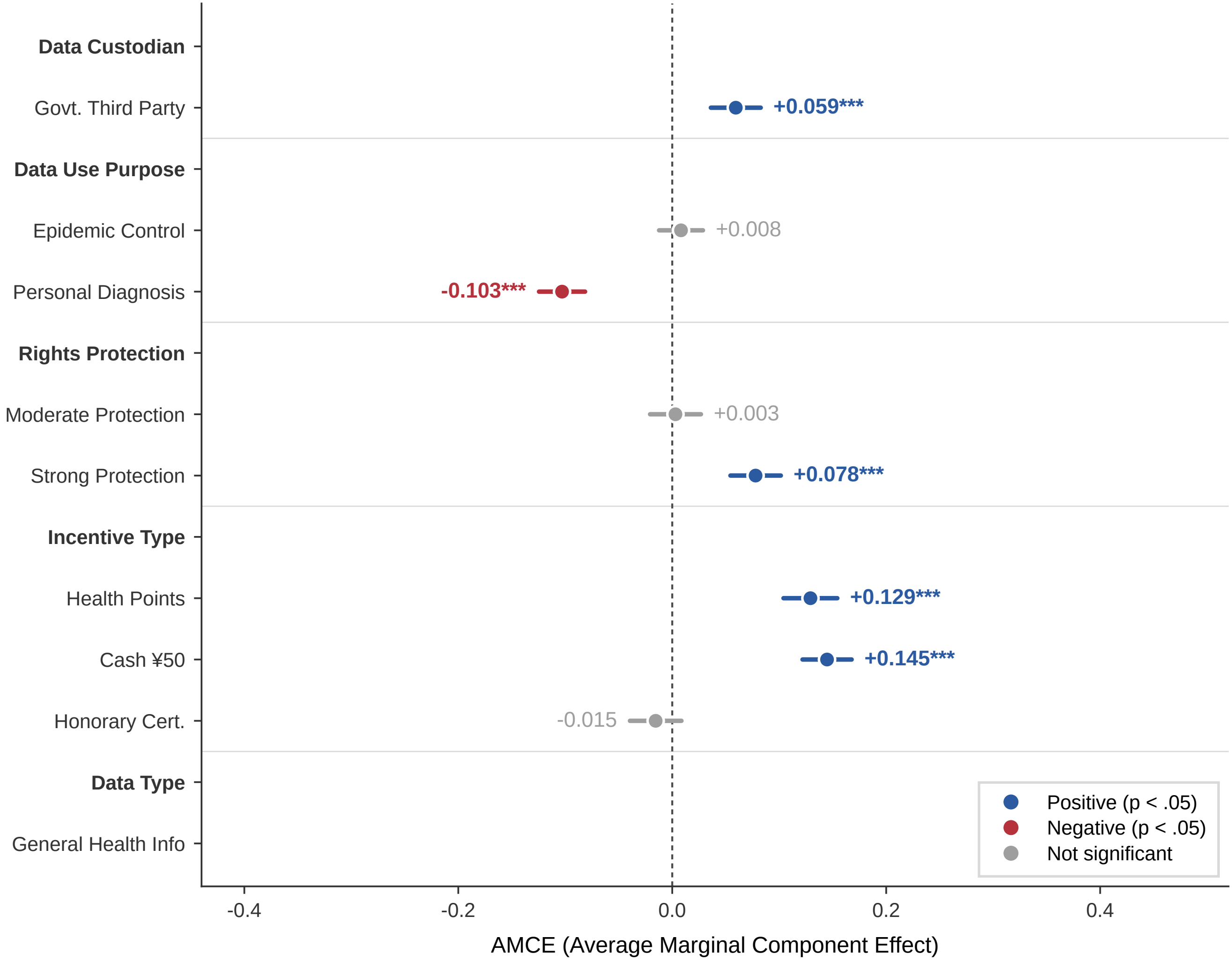

Note: *** p < .001, ** p < .01, * p < .05, † p < .10. Error bars = 95% CI.
