# Supplementary material for: Public preferences for health data sharing governance: a discrete choice experiment in China
Source: Front Public Health. 2026 Oct 20;14:1883099. doi: 10.3389/fpubh.2026.1883099 (PMC13727597; doi:10.3389/fpubh.2026.1883099)
Supplement: Supplementary file 2 [file Data_Sheet_2.ZIP › Reproducibility_Package/results/figures/amce_interaction_en.pdf]

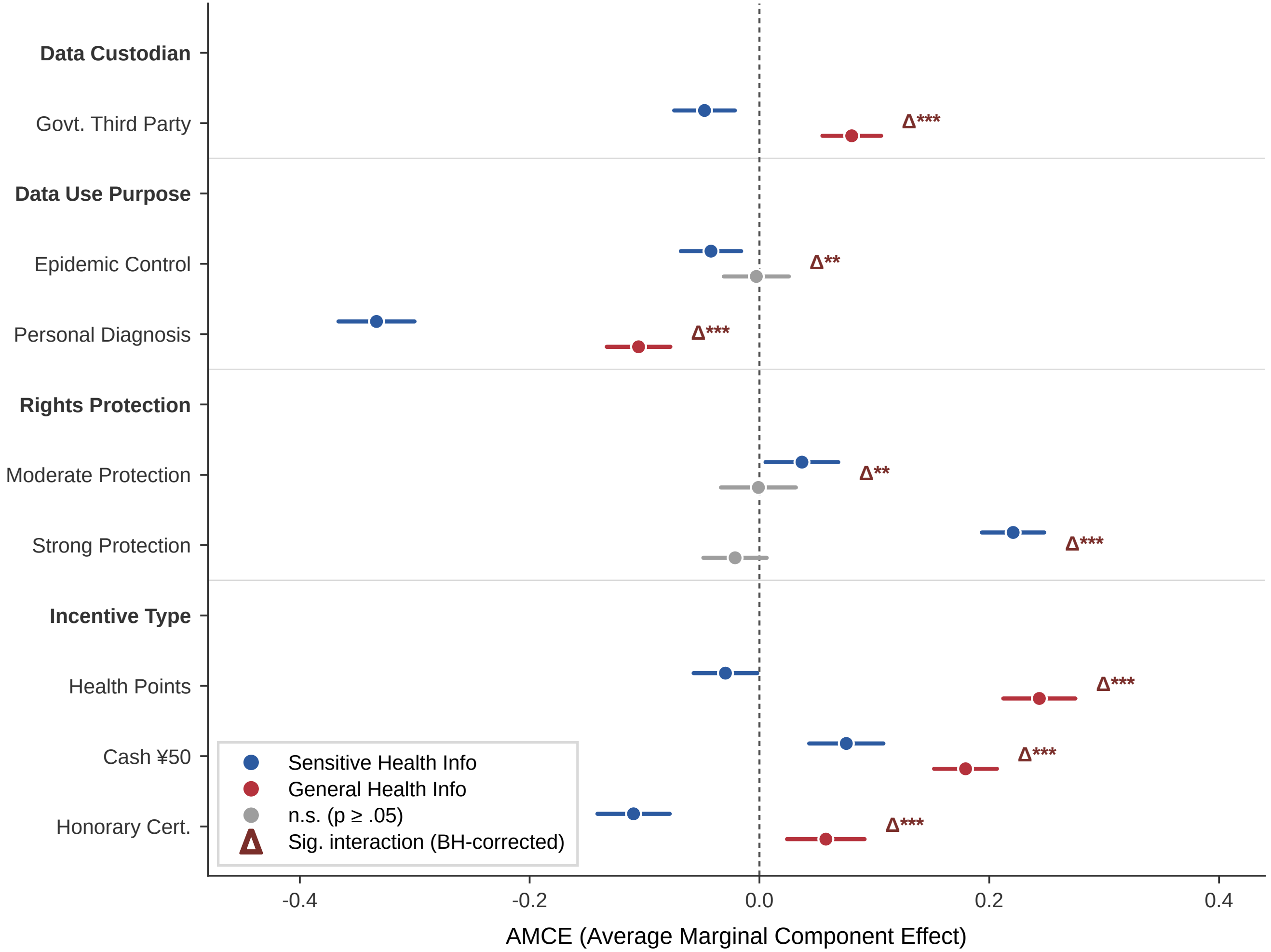

Note: Δ*** $p < .001$, Δ** $p < .01$, Δ* $p < .05$ (BH-corrected). Error bars = 95% CI.
