# Supplementary material for: Public preferences for health data sharing governance: a discrete choice experiment in China
Source: Front Public Health. 2026 Oct 20;14:1883099. doi: 10.3389/fpubh.2026.1883099 (PMC13727597; doi:10.3389/fpubh.2026.1883099)
Supplement: Supplementary file 2 [file Data_Sheet_2.ZIP › Reproducibility_Package/results/figures/heterogeneity_en.pdf]

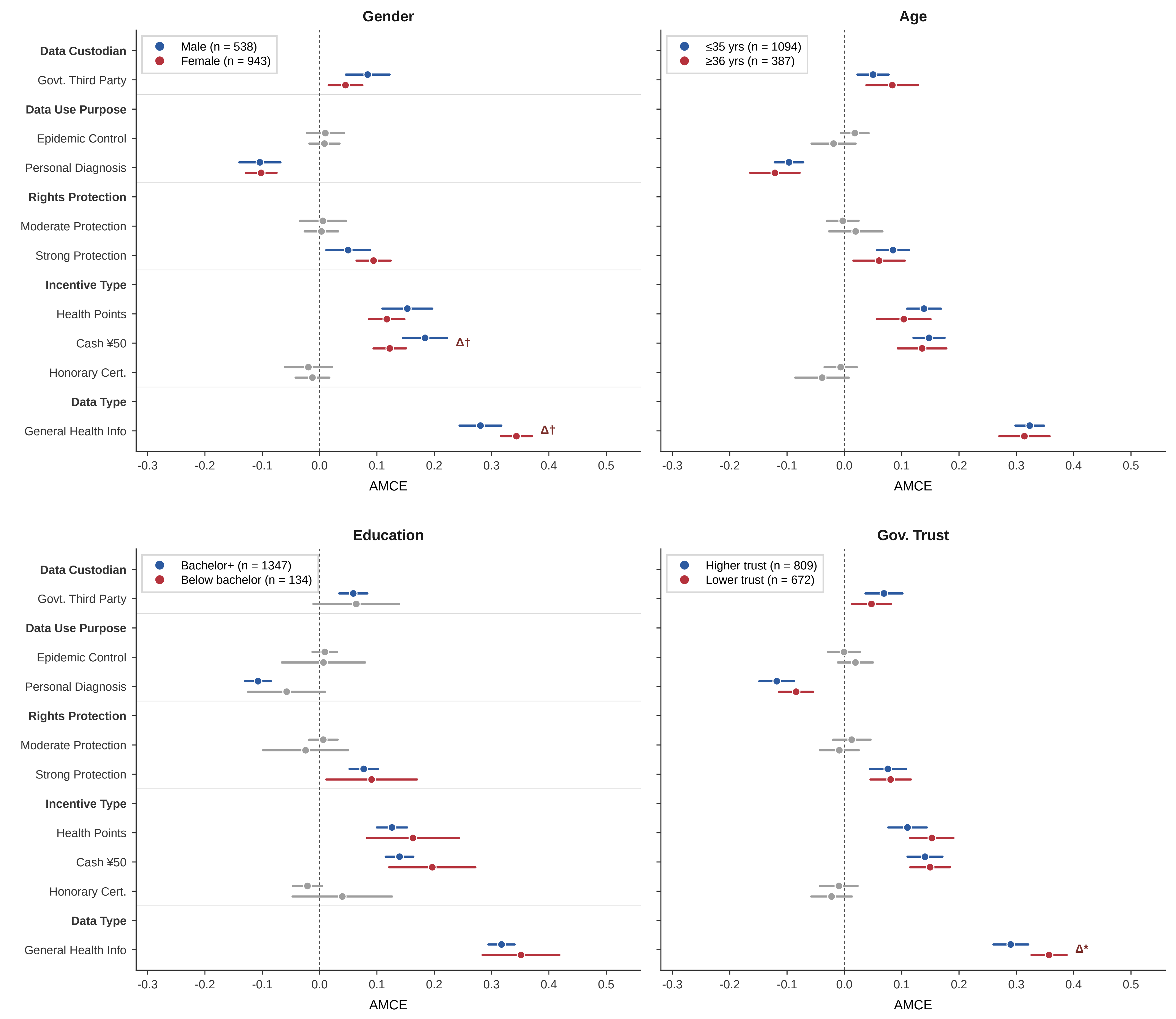

Note: Δ*** p < .001, Δ** p < .01, Δ* p < .05, Δ† p < .10 (BH-corrected between-group difference). Gray = n.s. Error bars = 95% CI.
